# Vegan versus meat-based pet foods: Owner-reported palatability behaviours and implications for canine and feline welfare

**DOI:** 10.1371/journal.pone.0253292

**Published:** 2021-06-16

**Authors:** Andrew Knight, Liam Satchell

**Affiliations:** 1 Centre for Animal Welfare, University of Winchester, Winchester, United Kingdom; 2 School of Environment and Science, Nathan Campus, Griffith University, Nathan, Queensland, Australia; University of Lincoln, UNITED KINGDOM

## Abstract

Consumer suspicion of conventional pet foods, along with perceived health benefits of alternative diets, are fuelling development of the latter. These include raw meat diets, *in vitro* meat products, and diets based on novel protein sources such as terrestrial and marine plants, insects, yeast and fungi. However, some claim vegan diets may be less palatable, or may compromise animal welfare. We surveyed 4,060 dog or cat guardians to determine the importance to them of pet food palatability, and the degree to which their animals displayed specific behavioural indicators of palatability at meal times. Guardians were asked to choose one dog or cat that had been within their household for at least one year, and not on a prescription or therapeutic diet. Of 3,976 respondents who played some role in pet diet decision-making, palatability was the third most important among 12 factors cited as important when choosing pet diets. For 1,585 respondents feeding conventional or raw meat diets, who stated they would realistically consider alternative diets, palatability was the fourth most important among 14 desired attributes. For the 2,308 dogs included, reported observations of 10 behavioural indicators of palatability at meal times reliably indicated significant effects of increased reports of appetitive behaviour by dogs on a raw meat diet, as opposed to a conventional diet. There was no consistent evidence of a difference between vegan diets and either the conventional or raw meat diets. For the 1,135 cats included, reported observations of 15 behavioural indicators indicated that diet made little difference to food-oriented behaviour. Based on these owner-reported behaviours, our results indicate that vegan pet foods are generally at least as palatable to dogs and cats as conventional meat or raw meat diets, and do not compromise their welfare, when other welfare determinants, such as nutritional requirements, are adequately provided.

## Introduction

The palatability of petfood is a matter of considerable importance, for both commercial and animal welfare reasons. In 2018, the global pet population was estimated as including 470 million dogs, and 370 million cats [1]. In 2014, pet food sales were estimated at Euro 131.7/USD 161.0 billion [2]. In 2019, US pet food and treat sales were valued at USD 36.9 billion [3] and the UK pet food market was expected to reach £2.8/USD 4.0 billion by the end of the year, having risen 17% over the previous five years [4]. (Using USD equivalents from May 2021).

A market of such size drives considerable research and product development, and between January 2013 and October 2014, over 6,000 new petfood products (3,000 dry and 3,200 wet pet foods), as well as 4,000 new pet snacks, were launched globally [5 in 6]. Some of the new products being developed include raw meat diets and *in vitro* meat products, and diets based on other novel protein sources, including terrestrial and marine plants, insects, yeast and fungi. Conventional animal-based protein sources have supply limitations, exacerbated by increasing food and land use due to a growing human and pet population. Increased ‘humanization’ of pets is also resulting in increased feeding of ‘premium’ pet foods, using greater proportions of human-grade ingredients and further contributing to this problem [7]. Significant concerns now exist regarding the environmental sustainability of animal agriculture and those pet foods reliant on animal products [8–10]. A significant proportion of consumers are also suspicious of conventional pet foods, and perceive that alternatives may provide a more natural feeding experience, or health benefits [11]. Vegetable ingredients within alternative diets may have composition and macronutrient/micronutrient digestibility comparable to conventional animal-derived ingredients [12–14]. Such factors have encouraged the development of alternative diets with the goal of maintaining nutritional soundness, potentially providing health and environmental benefits, and providing good palatability [15].

However, concerns exist that the imposition of human petfood preferences may be detrimental to the welfare of pets. Noting the biologically carnivorous nature of cats, Zoran and Buffington [16] asserted that “many of the chronic health problems of domestic cats are directly or indirectly related to nutrition or lifestyle changes that have been imposed on them by their owners”. In the UK, Loeb [17] claimed (albeit without evidence) that “… an owner who feeds his or her cats a vegan diet … could be committing a crime under the Animal Welfare Act …”, and has repeated similar claims elsewhere [18].

### Welfare provisions affected by pet food

Do vegan diets diminish the welfare of dogs and cats? Building on the original Five Freedoms of the UK Brambell Committee [19], the updated Five Domains Model [20] and the European Welfare Quality assessment system, modern conceptualisations of good animal welfare require that animals be provided with the ‘Five Provisions’ of good nutrition, a good environment, good health, opportunities to engage in appropriate natural behaviours, and positive mental experiences [21]. It is also considered important that animals be provided with not only the opportunity to experience ‘a life worth living’, but ‘a good life’ (meaning a good quality of life—QoL). This is defined as one in which the overall balance of positive experiences, significantly outweighs negative experiences [22].

With respect to the Provisions of nutrition and health, clearly, diets need to be nutritionally sound to ensure good health and welfare. Nutritional requirements vary with species, life stage, and physiological status. The nutritional suitability of vegan diets is a sizeable topic, meriting studies in its own right. These are outwith the scope of this article, but are addressed elsewhere (e.g. [23–30], and forthcoming studies by the lead author and colleagues).

Environments are not normally affected by diet, other than through faecal contamination, which can be a concern in its own right [8]. However, the remaining Provisions of behaviour and mental experiences could be affected by diet. When considering QoL acceptability, Mellor [31] asks “How will expressions of normal behaviour be encouraged and harmless wants met?” The feeding regime of modern, domesticated dogs and cats often bears little resemblance to natural feeding behaviour [32]. Dogs co-evolved with humans to participate in hunts of prey species and scavenge around camp fires and early human settlements [33]. Cats evolved to hunt a wide variety of mammals, birds and insects. The timing of kills, and subsequent food intake, were largely unpredictable [16, 32]. In contrast, modern, domesticated dogs and cats are usually fed diets based on body parts, often from farmed animals they would not naturally consume, or wild-caught fish, formulated with a variety of ingredients and processing methods they would not naturally encounter, packaged in tins, cartons, and pouches, or as kibble. They are fed at predictable times daily, or *ad libitum* (i.e. with food always available), in the case of kibble [16, 34–36]. Whilst acknowledging some level of ongoing hunting behaviour in cats, most domesticated dogs and cats do not rely on hunting to source the majority of their food. These deviations from natural feeding behaviour are significant, and occur regardless of supplied pet food type.

When considering how the acceptability of pet welfare varies with pet food type, this leaves only the Provision of positive mental experiences to consider. Mellor [31] asks “… what provisions have been made to ensure that consuming the food provided will be an enjoyable experience?” Some concerns exist that vegan pet foods may be less enjoyable. Brown [37], for example, asserted that diets lacking animal-based ingredients would be less palatable. With respect to food, rewarding properties encompass taste, odour, sight, and texture (particularly, ‘mouthfeel’), as well as the act of eating itself [6, 38]. Such factors determine the palatability of food [39], which is the extent to which it is enticing to animals, encouraging consumption [6].

### Pet food palatability

A range of food-seeking behaviours and indicators of dietary palatability have been described for dogs and cats. For dogs, these include rapid consumption of food, vocalising for food, stealing food, raiding bins, waking the owner during the night for food, staying near the food bowl, or being aggressive over food [40]. Callon and colleagues [41] used factors such as tail wagging, licking air/lips, licking ground or bowl after consumption, pushing face through kennel bars, and jumping at the front of kennels. By using puzzle feeders, Thompson and colleagues [42] concluded that canine food preference could be confidently and reliably inferred from behaviours such as investigation and sniffing. Di Donfrancesco and colleagues [43] noted that suspicion or caution with respect to novel food can manifest as sniffing at food, or lack of signs of interest.

For cats, in a study of wet diets, Van den Bos and colleagues [44] found that licking or sniffing the food bowl, lip licking and face grooming, were indicators of good palatability, whereas sniffing food itself, or nose licking, indicated some aversion for offered food. Savolainen and colleagues [45] also found that lip licking was more frequent with preferred food, and Becques and colleagues [46] similarly found that cats spent significantly longer sniffing at food less preferred, although only during initial exposure. They found no differences in latency to eat, or speed of consumption. Savolainen and colleagues [45] also identified as signs of lower food preference, backward ear flicking, nose licking, tail flicking and body grooming. Hanson and colleagues [47] found that good palatability was indicated by nose licking, tongue protrusion, lip-smacking and increased duration of ‘half closed eyes’.

Such behavioural indicators often have sound biological rationales. Olfaction, for example, plays multiple roles, prior to and during feeding. It aids with location of food (the sense of smell is particularly strong in dogs and cats), assessment of freshness, rancidity or possible toxicity, and may stimulate appropriate digestive and salivary secretions, and gastrointestinal activity [6]. In the cases above, increased sniffing at food less preferred, may indicate a perceived greater need to investigate it, prior to consumption.

However, assessment of behavioural indicators may be complex. Tobie and colleagues [6] reported that feline sniffing and licking behaviour did not vary with palatability, when dry food was offered, in the ways that it did for wet food.

The evaluation of such indicators of palatability is essentially acceptability (or ‘one pan’) testing—one of two main methodologies employed by the petfood industry. The other main methodology utilised is preference testing (or ‘two pan’), in which a choice is provided between two diets presented simultaneously [6].

Limited published studies exist comparing the palatability of meat- versus plant-based diets, and exploring the effects of processing, such as cooking time, and application of fats or supplements. When investigating the role of olfaction, Houpt and colleagues [48] found dogs preferred meat-based diets to those composed of maize and soybean meal. In a study of eight kennelled beagles, offered both meat- and plant-based diets, Callon and colleagues [41] found no significant difference in palatability indicators such as level of anticipation pre-consumption, hesitation, time to consume feed and ease of distraction. They found that level of interest post-consumption was highest when dogs consumed the animal-based ingredients. However, they were uncertain whether this was because of palatability, or because the animal-based diets were less satiating, with dogs seeking more food after feeding. Keller [49] found that animal-based proteins have lower satiety ratings than plant-based proteins.

Alegría-Morán and colleagues [50, 51] analysed 1,771 preference tests conducted on 34 kennelled dogs, and 1,021 preference tests on 24 kennelled cats. They found that food preferences in both species were negatively correlated with crude fibre and dry matter levels. This is predictable, given that dietary preferences depend on food sensorial qualities, which are partly determined by nutrient composition and formulation. Small particle size and increased moisture content facilitates the liberation of desirable volatile compounds [52, 53]. Conversely, de Brito and colleagues [54] found that increased dietary moisture did not affect canine food preference. Decreased water content facilitates storage, reducing the possibility for contamination with mould or other pathogens [55]. Accordingly, the petfood industry seeks to enhance palatability of dry foods without increasing moisture content, through utilisation of highly digestible ingredients, natural or artificial umami flavours (which usually indicate high amino acid concentrations; [56]), and/or an external palatable fat cover [50], although this can increase obesity risks [57].

Palatability studies normally use quite limited animal numbers. Pires and colleagues [58] systematically reviewed 16 papers describing palatability studies using cats. Sample sizes varied from nine to 60. To date, no large scale study of dogs or cats has been published, describing how palatability indicators vary with main diet type—whether conventional meat, or alternatives such as raw meat, *in vitro* meat, insect- or yeast-based, vegetarian or vegan diets. Accordingly, we designed a study to explore the relative palatability of such different diets. Our null hypothesis was that canine and feline palatability indicators would not significantly vary with diet type.

The success of new pet foods depends not only on palatability, but on nutritional value, and the views of consumers. In 2019, Schleicher and colleagues [59] reported that 2,181 surveyed respondents rated “pet preference” as the sixth most important among 25 determinants of their pet food purchasing decisions. Their study was distributed through US-based networks, and a large proportion (564/1975, 28.6%) were employed in the veterinary or animal care industries. In 2021, Dodd and colleagues [29] reported that among 1,025 cat guardian respondents to a survey, palatability was rated as equal third most important of 10 determinants. Their study was distributed through Canadian and US-based networks. We sought to determine the importance of palatability as a purchasing determinant, to a broader, more international demographic.

## Methodology

We designed an online survey for dog or cat owners. Owners were asked about factors, including palatability, they considered important when choosing pet foods. They were also asked to choose one dog or cat that had been within their household for at least one year, and not on a prescription or therapeutic diet.

Exclusion of animals maintained on therapeutic diets was undertaken for two reasons. First, most pets are not maintained on therapeutic diets, and the primary objective was to investigate possible effects on health and behavioural outcomes, of conventional diets, and common alternative diets. Second, many animals maintained on therapeutic diets are likely to have dietary-responsive health conditions, and alterations in health status may sometimes alter food-associated behaviours [60]. We aimed to minimise inclusion of animals whose behavioural or health outcomes might be affected by such diets. Hence owners of animals on therapeutic diets were asked to consider the non-therapeutic diet in use prior to the commencement of the therapeutic diet, and to report on food-associated behaviours observed with that previous, normal diet.

Owners were asked about the main ingredients within their pet’s normal diet. Pet food options provided included conventional, raw and *in vitro* meat-based diets, insect-, fungi- and algal-based, and vegetarian, vegan or ‘other’ diets. Vegetarian diets were explained to respondents as including eggs or milk, but not meat, and vegan diets as eschewing any animal products.

Owners were advised at the start of the section on behavioural questions, to focus on “your animal’s reaction to their normal meals (not snacks).” Survey questions asked about owner perceptions of the prevalence of specific palatability indicators associated with meals. These were 10 canine and 15 feline behaviours described in previous literature as definitely or possibly indicating palatability. Owners were asked about the extent to which these behaviours were displayed, using a five point Likert scale from Strongly Disagree (1) to Strongly Agree (5).

### Potentially confounding factors

We considered a range of factors that could potentially alter behavioural responses to food. Pets in multi-animal households may compete with one another for food, which has significant potential to alter behavioural indicators, such as latency to approach food, time spent investigating food, and consumption kinetics. Stress, including social stress, also has potential to alter pleasure perception and hence food preference, in some mammals [61, 62]. To account for these factors, we asked whether pets normally compete for food at meal times, and excluded such pets from further analysis.

Snacks and treats between meals may reduce enthusiasm for meals, but we chose not to exclude animals who received treats regularly, expecting most to receive such treats. Unlike dogs, the feeding strategy of cats would naturally involve the frequent consumption of small meals comprised of prey animals [16]. Many cats are also fed *ad libitum*. This could mean that cats are more likely to leave such food and return to it later. To investigate the possible effect of this, we also sought to determine the proportion of cats fed *ad libitum*.

Exercise levels can significantly affect calorific requirements, and hence, could affect behavioural responses to food. Dogs who only walk on the lead or are likely to be active for up to one hour daily have lower calorific requirements than those with higher activity levels [63]. Hence, we determined whether palatability indicators were different for dogs and cats exercising more, or less than, one hour daily.

Climate seasonality may also affect appetite, and in conflicting directions. Serisier and colleagues [64] found that cats consume less in winter, possibly due to negative effects on exercise levels. Conversely, Alegría-Morán and colleagues [51] found that feline food intake increased, especially in females, in winter. Similarly, both dogs and poultry were found to have increased food intake during winter, probably due to decreased calorific need to maintain body temperature [65–67]. We preferred neither summer nor winter, but aimed to at least keep seasonality consistent. Our survey was timed to correspond to the northern hemisphere summer months, and the southern hemisphere winter.

A very young, or advanced, age, could conceivably affect behavioural responses to food. Hence, we determined mean ages within each of the main dietary groups studied, and the extent to which diet groups and reported behaviours varied with age. Given increased interest in some alternative pet foods in recent years, we expected animals maintained on such diets might be younger.

Sex and neuter status could affect hormonal status, and conceivably metabolic rate, altering calorific needs, and hence behavioural responses to food. Hence, we determined proportions of animals in sex/neuter groups, and the extent to which diet groups and reported behaviours varied with sex/neuter status.

Alegría-Morán and colleagues [50] found that canine food preferences were unaffected by dog weight and season—although these factors affected level of food intake. Breed did affect preference. However, the effects of breed can be unclear. For example, brachycephalic breeds, such as the Boxer, have a lower olfactory capacity due to morphological limitations, which affect the capacity of their nasal anatomy, and the position of their olfactory lobes [68]. These changes could be expected to affect their food preferences. As a dolichocephalic breed, German Shepherds could be expected to exhibit opposite effects. And yet, Hall and colleagues [69] demonstrated that Pugs outperformed German Shepherds in a test of odour discrimination. We were also concerned that small numbers within breed groups would limit our ability to statistically analyse subsequent results, and so ultimately elected not to discriminate by breed within this study.

### Survey pilot and distribution

We created an online survey using the ‘Online surveys’ platform (https://www.onlinesurveys.ac.uk). This complies with the UK General Data Protection Regulation, following the UK Data Protection Act 2018, and was used by 88% of UK higher education institutions by 2019 [70], including our University of Winchester.

We piloted our survey to 25 respondents in April 2020. Improvements were then made to both survey structure and questions. With respect to structure, changes were made to the ordering of survey parts, to minimise inadvertently biasing answers to questions about palatability or health. These survey sections were moved toward the beginning, to eliminate chances that answers might be affected by prior answers about dietary choices, particularly where unconventional diets were used, e.g. if an owner reporting use of an unconventional diet consciously or unconsciously subsequently downplayed any palatability or health problems.

Similarly, questions about palatability indicators were positioned prior to those about body weight, to avoid similar problems. For example, an owner falsely believing and reporting their animal as not overweight, might then under-report signs of excessive food intake, due to a desire to be consistent with their previous answers.

Similarly, changes were made to the ordering of questions about animal location (e.g. indoors vs. outdoors) and activity level, and veterinary opinions about animal health, some of which are the subject of related, forthcoming studies. In general, the variable most likely to be dependent, was shifted prior to any corresponding independent variable. Various questions were also clarified and simplified.

The final survey was made available from May—December 2020. It was widely advertised through social media to dog and cat interest groups. Paid Facebook advertising and several volunteers were utilised to increase survey exposure. Facebook advertising demographics were unlimited, other than to include terms relating to dogs and cats. In anticipation of lower levels of unconventional diets, and the need to achieve group numbers sufficient for statistical analysis, volunteers and the authors tried to reach unconventional pet food interest groups, as well as conventional dog and cat interest groups. However, by careful wording choice, no bias for or against any particular diet was implied within advertising materials, or within the survey questions or explanatory text.

### Statistical analysis

We chose software package Jamovi [71] in preference to other choices partly because it is open, transparent, runs the R syntax programming language for statistics, and because all its analyses are fully reproducible. With a sample size as large as ours, effects could be significant using traditional null-hypothesis significance testing cut offs (*p* < 0.05), but not *meaningfully* different. For optimal discussion of these results to meaningfully inform understanding of the behavioural effects of differing pet diets, we used conservative p value criteria (of *p* < 0.001), but primarily drew inference from effect sizes throughout. We used the oft-cited heuristics provided by Ferguson [72], to describe the effect sizes.

We investigated the impact of three main diet types identified, on 10 canine and 15 feline behavioural indicators of palatability. The principal evaluation of our research question was conducted using three-level one-way ANOVA as we had categorical independent variables and scale dependent variables. These ANOVA were reported with the test statistic *F*, a *p* value for tests of significance, and we used generalised ω^2^ as our measure of effect size. Due to our large sample size, and thus liberal threshold for ‘statistical significance’, we primarily drew inference about the meaningfulness of effects using ω^2^ (the merits of which have been discussed elsewhere; e.g. [73]). This prioritises reasonable inference about the degrees of difference between groups, more than the binary state of significant or not. Regardless of the significances of omnibus ANOVA tests comparing all three diet groups, we conducted pair-wise comparisons of the diet conditions for completeness of reporting. These tests were best conducted using a t test statistic, *p* values and again, inference was prioritised on Cohen’s *d* measures of effect size. In cases where we tested categorical variables as sources of variation in other categorical variables (such as diet and activity category), we drew inference from χ^2^ tests, reporting the test statistic, *p* values and Cramer’s *V* measure of effect size. When evaluating the effects of age on behaviours, where we had two dimensional variables, we used Pearson’s *r* correlations.

We also examined covariation between the behaviours reported using factor analyses. These were ‘oblimin’ exploratory factor analyses. That is, they are data-up (rather than researcher-defined) groupings of common covariation between measures in the data, and these factors are allowed to share some variance with each other in an oblique solution.

When discrete patterns of behavioural indicators were identified, these were assessed as positive, negative or indeterminate palatability indicators, based on knowledge of canine or feline behaviour, and previous studies of palatability indicators.

### Ethical approval and data availability

Our research complied with the University of Winchester Ethics Policy [74] (approval reference RKEEC200304_Knight). Our data analysed are accessible at https://rebrand.ly/2020/pet-food-consumers-study.

## Results

### Overall results

#### Human participants

Of 4,060 respondents, 4,057 confirmed they met the survey conditions (18 years or older, with answers relating to one dog or cat resident within their household, for at least one year). Females comprised 90.7% (3,681) of respondents. Most age brackets from 18 to 70+ were well represented, other than extreme ends where numbers were low. The overwhelming majority identified their geographical region as the UK (71.0%, 2,882) or Europe (16.7%, 677), with North America (5.4%, 218) and Australia/New Zealand/Oceania (3.9%, 160) being the next most prevalent continental regions. In total, 94.2% (3,820/4,057) of respondents reported they were from the Northern hemisphere. Six hundred and twenty-nine (15.5%) worked in the pet or veterinary industries. Respondents followed a variety of diets themselves, with the most common being omnivorous (38.6%, 1,566), vegan (23.6%, 958), reducetarian (omnivore reducing animal product consumption) (885, 21.8%), vegetarian (10.2%, 412) and pescatarian (consuming fish but no other meats) (5.1%, 206).

#### Importance of palatability to owners

Four thousand and nine respondents indicated their level of involvement in choosing their pet’s diet. Primary decision-makers comprised 95.0% (3,807), those who played some lesser role comprised 4.2% (169), and those who played no role comprised 0.8% (33). Those 99.2% (3,976) playing at least some role, were asked which factors were important when choosing pet diets. Palatability (described as how much the pet seems to ‘like’ its food), was the third most important among 12 cited factors, (including ‘other’ but excluding ‘none’). Palatability was cited by 69.5% (2,765 respondents), after health and nutrition (91.1%, 3,624) and diet quality (70.4%, 2,798).

All participants were asked about the main ingredients within their pet’s normal diet. The importance of palatability was also highlighted by the 2,514 respondents who used a conventional meat formulation as their pet’s normal diet, and the 894 who used a raw meat formulation. Of these combined 3,408 respondents, 46.5% (1,585) stated they would realistically choose diets not based on conventional or raw meat, if such alternative diets offered their desired attributes and standards. Palatability was the fourth most important among 14 desired attributes (including ‘other’) the alternative diet would need to provide, that was cited by these 1,585 respondents. It was cited by 70.5% (1,117 of these respondents), after confidence about pet health, and confidence about nutritional soundness (both 82.6%, 1,309), and good quality (72.4%, 1,147).

#### Dog and cat diets

Initially included were 2,639 dogs and 1,418 cats (total: 4,057). After initial examination of their diets, we focused on animals maintained on three main diets: conventional meat, raw meat, and vegan pet food. Seventy six ’other’ diets were examined and reclassified into conventional meat, mixture or unsure, depending on further details provided in textual answers.

Several smaller dietary groups were excluded from further analysis, as described under dog and cat diets respectively, to avoid potentially substantial differences in variances between dietary groups of small and larger sizes, which could adversely affect our statistical analysis. Some smaller groups were also excluded due to lack of clarity concerning main ingredient type, or current unavailability of these sources as pet maintenance diets (as distinct from treats, snacks or supplements).

As mentioned, we chose not to exclude animals who received treats regularly, expecting most would fall within this group. This turned out to be true, with 2,562 (63.9%) of 4009 participants answering this question, providing treats at least once daily.

### Canine results

#### Dog diets

Two thousand, six hundred and thirty nine dog owners responded, each describing a single dog. Of these, 2,612 indicated the main diet their dog was maintained on. The three major dietary groups reported were conventional meat (1,370), raw meat (830) and vegan (336). In total 2,536 dogs were jointly maintained on these three diets, with small numbers on vegetarian diets (35), laboratory-grown meat (7), insect (6) and fungi-based diets (1). The remainder reported using mixtures (17) or were unsure (10).

**Fig 1:**
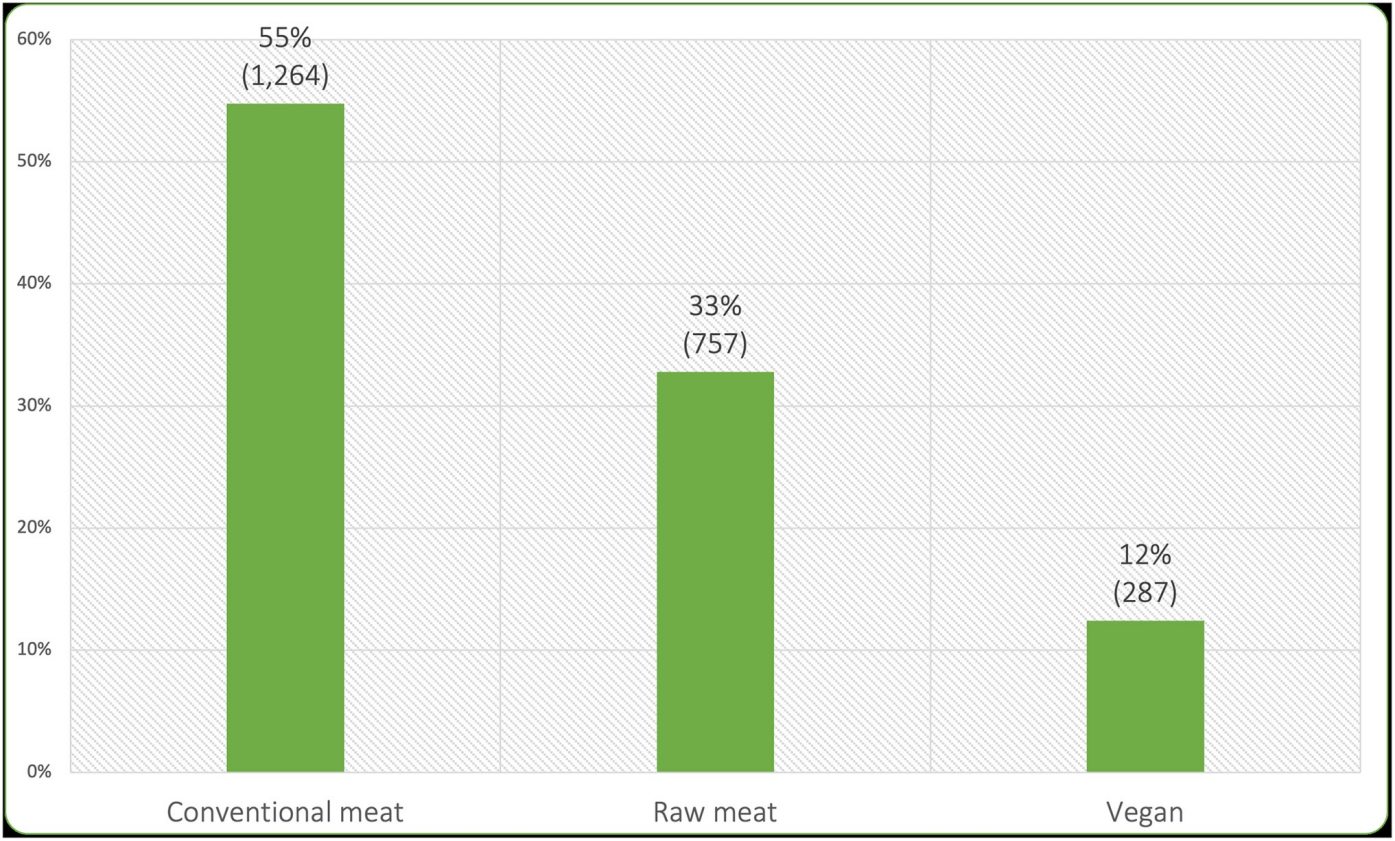
Diets of 2,308 sampled dogs.

Owners were asked whether their dogs normally competed with any others at mealtimes. After eliminating 228 ‘yes’, ‘unsure’ or ‘blank’ answers, 2,308 dogs remained in the three main dietary groups who reportedly did not compete for food (Fig 1). These 2,308 dogs comprised the sample subjected to further analysis.

#### Sex/neuter status and age

**Fig 2:**
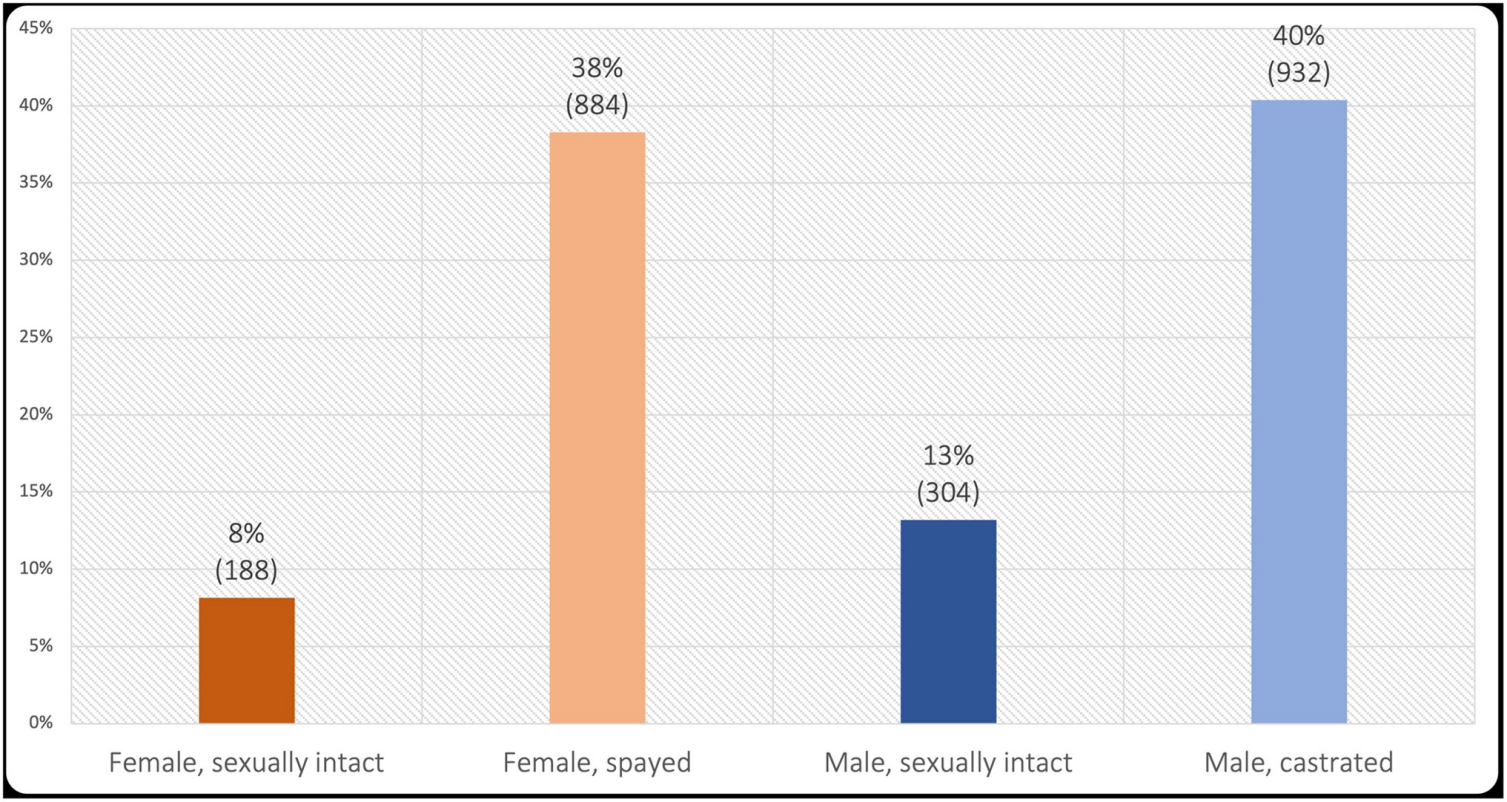
Sex/neuter status of 2,308 sampled dogs.

The sex/neuter status of these 2,308 dogs is provided in Fig 2. Neutered animals (those spayed or castrated) comprised 82% of the females, and 75% of the males. There was a small difference in sex/neuter status by diet type (χ^2^(6) = 52.30, *p* < 0.001, Cramer’s *V* = 0.11), explained by sexually intact male and female dogs being overrepresented in raw meat diets and underrepresented among other diet types. But it should be noted how small this effect was—it was smaller than Ferguson’s recommended practical significance size. Similarly, the differences in palatability behaviours between sex/neuter groups were negligible (all *F* ≤ 6.86, all ω^2^ ≤ 0.01). Overall, there was no notable evidence that sex/neuter status affected diet type or owner-reported behaviours.

Between each of the three main dietary groups studied, there was a significant difference in dogs’ mean ages (*F*(2, 2303) = 30.60, *p* < 0.001, ω^2^ = 0.03). Vegan dogs were the oldest (*M* = 7.26, *SD* = 3.65) compared to dogs on conventional meat (*M* = 6.35, *SD* = 3.70, *t*(2303) = 3.85, *p* < 0.001, *d* = 0.25) and raw meat (*M* = 5.44, *SD* = 3.36, *t*(2303) = 7.31, *p* < 0.001, *d* = 0.51) diets. Further, in this sample, the conventional meat-based dogs were older than the raw meat-based dogs (*t*(2303) = 5.55, *p* < 0.001, *d* = 0.26). Age did not meaningfully relate to any of the owner-reported behaviours with all correlations being negligible in size (all *r* ≤ |0.07| with the largest being jumping behaviour).

#### Canine palatability indicators

Of principle interest was the behavioural reaction displayed by the dogs when they were provided with meals. The owners were asked to report the extent to which they thought their dog displayed 10 behaviours (on a scale of Strongly Disagree (1) to Strongly Agree (5)). Results for the 10 canine palatability indicators in are provided in the S1 Appendix. Lack of reporting in some cases reduced total numbers.

#### Effect of activity level

We explored the potential effects of dogs’ activity levels on their food-orientated behaviours. We categorised dogs’ activity levels into those which typically spent more than 60 minutes daily being active (‘active’ dogs *n* = 1480) and those which spent less than 60 minutes daily being active (‘sedentary’ dogs, *n* = 828).

In a chi-square test of independence, there was a small difference between the frequency of active and sedentary dogs in the diet groups (χ^2^(2) = 8.20, *p* = 0.017, Cramer’s *V* = 0.06). This was explained by a slight difference between diets, where dogs on vegan diets were more likely to be sedentary (43.20% of vegan dogs) than those fed conventional meat (35.40%) or raw meat (33.80%) diets. We compared the activity groups for differences in food-oriented behaviour. We used a series of t tests to compare the activity groups, and we inferred significance from a multiple comparison-corrected conservative significance of *p* < 0.001. Only jumping (*t*(2304) = 3.75, *p* < 0.001, *d* = 0.16) and vocalisation (*t*(2304) = 3.64, *p* < 0.001, *d* = 0.16) behaviours were significantly different by these standards. We further tested for interactions between activity category and diet group using 3 (diet) x 2 (activity) ANOVA on food-oriented behaviours. Of the 10 possible effects, none met a conservative multiple-comparison correction of *p* < 0.001. The largest effect was the small interaction for salivating behaviours (*F*(2, 2300) = 15.56, *p* = 0.017, ω^2^ = 0.00) and all other effects were smaller (*F* ≤ 8.97, *p* ≥ 0.088, ω^2^ ≤ 0.00). Overall, activity level minimally impacted food-oriented behaviour.

#### Dimensions of behaviour

There was overall consistency in the ratings of food-oriented behaviours for the dogs in the sample. That is, if a dog was reported as more frequently displaying one of the food-oriented behaviours, they were likely to display the other behaviours, as was shown with the reliability coefficient McDonald’s ω = 0.76 (see [75]). For this analysis ‘sniffs food’ was reverse scored, as a first-pass consistency analysis indicated this dimension was inverse to the others (as food-oriented behaviour increased, ‘sniffs food’ decreased). For all following analyses ‘sniffs food’ is forward scored.

Beyond overall consistency, we investigated the patterns of reporting food-oriented behaviour using factor analysis. Parallel analysis of oblimin exploratory factor analysis models suggested there were four overall factors that satisfactorily explained the reports of dogs’ behaviour (RMSEA = 0.05 [0.04, 0.06], TLI = 0.97). One of these factors describes general enthusiasm—dogs who were reported to eat quickly (loading = 0.69) were those who approached the food more quickly (0.83), wagged their tail more (0.43) and were less likely to sniff their food (-0.69). Distinct to this were the energetic behaviours, with dogs who were reported to jump (0.73) more often also being those who also vocalised (0.72) more often. There was a food defensive factor, explained by variance in staying near the food bowl (0.61) and guarding the food (0.47). Finally, there was a factor describing anticipation with similar ratings of licking lips (0.92) and overtly salivating (0.40).

The four discrete behavioural patterns identified were assessed as positive or negative palatability indicators, in this sample of 2,308 dogs, based on knowledge of canine behaviour, and on previous studies of canine palatability indicators [40–43] (Table 1).

**Table 1:** Behavioural indicators of positive or negative palatability in 2,308 sampled dogs.

**Positive (9)**	eats quickly, approaches food quickly, wags tail, jumps, vocalises, salivates, licks lips, stays near food bowl, guards food
**Negative (1)**	sniffs food

#### Tests of dietary difference

Table 2 reports on the distribution of the reports of the dogs’ food-oriented behaviour, with Table 3 reporting inferential tests of differences by diet. The average response across the diet was typically in the same response range, and in the inferential tests, there were small differences between the diet types. The between diet comparisons showed an average Cohen’s *d* of |0.07| which is smaller than oft-cited recommended minimum effects (*d* = 0.40; [72]). Whilst a large sample size liberalises estimates of significance, the results presented in Table 3 reliably demonstrated small effects of increased appetitive behaviour by dogs on a raw meat diet as opposed to a conventional diet. There was no consistent evidence of a difference between vegan diets and either the conventional or raw meat diets.

**Table 2:** Descriptive statistics of the owner reported food-oriented behaviours, with range Strongly Disagree (1) to Strongly Agree (5).

Factor	Variable	Diet		
		Conventional Meat	Raw Meat	Vegan
		Mean (SD)	Mean (SD)	Mean (SD)
Enthusiasm	Eats Quickly	3.49 (1.35)	3.84 (1.20)	3.69 (1.23)
Enthusiasm	Approach	3.88 (1.30)	4.31 (1.03)	4.17 (1.02)
Enthusiasm	Wag tail	3.87 (1.28)	4.22 (1.09)	3.99 (1.20)
Enthusiasm	Sniffs Food	2.99 (1.35)	2.88 (1.38)	3.06 (1.29)
Energetic	Jump	2.55 (1.41)	2.91 (1.50)	3.86 (1.44)
Energetic	Vocalise	2.29 (1.31)	2.52 (1.44)	2.33 (1.40)
Anticipation	Salivate	2.80 (1.36)	3.31 (1.37)	3.00 (1.48)
Anticipation	Licks lips	3.34 (1.41)	3.78 (1.29)	3.61 (1.30)
Defensive	Stays near bowl	3.28 (1.30)	3.51 (1.30)	3.40 (1.28)
Defensive	Guards food	1.88 (1.17)	1.82 (1.11)	2.22 (1.26)

Note: ‘-Enthusiasm’ denotes a variable inversely related to ‘Enthusiasm’.

**Table 3:** Comparison between diet groups for the owner-reported food-oriented behaviour.

Variable	Difference between all three diets	Conventional vs Raw	Conventional vs Vegan	Raw vs Vegan
Eats Quickly	*F* = 17.15, ω^2^ = 0.01***	*t* = -5.80 *d* = -0.12***	*t* = -2.24 *d* = -0.05	*t* = 1.64 *d* = .03
Approach	*F* = 32.93, ω^2^ = 0.03***	*t* = -7.92 *d* = -0.17***	*t* = -3.78 *d* = -0.08***	*t* = 1.69 *d* = 0.04
Wag tail	*F* = 19.37, ω^2^ = 0.02***	*t* = -6.22 *d* = -0.13***	*t* = -1.51 *d* = -0.03	*t* = 2.70 *d* = 0.06
Sniffs Food	*F* = 2.38, ω^2^ = 0.00	*t* = 1.76 *d* = 0.04	*t* = -0.77 *d* = -0.02	*t* = -1.90 *d* = -0.04
Jump	*F* = 16.21, ω^2^ = 0.01***	*t* = -5.34 *d* = -0.11***	*t* = -3.32 *d* = -0.07	*t* = 0.41 *d* = 0.01
Vocalise	*F* = 6.99, ω^2^ = 0.01***	*t* = -3.71 *d* = -0.08***	*t* = -0.51 *d* = -0.01	*t* = 1.98 *d* = 0.04
Salivate	*F* = 32.29, ω^2^ = 0.03***	*t* = -8.04 *d* = -0.17***	*t* = -2.22 *d* = -0.05	*t* = 3.24 *d* = 0.07
Licks lips	*F* = 25.96, ω^2^ = 0.02***	*t* = -7.10 *d* = -0.15***	*t* = -3.04 *d* = -0.06	*t* = 1.84 *d* = 0.04
Stays near bowl	*F* = 7.26, ω^2^ = 0.01***	*t* = -3.78 *d* = -0.08***	*t* = -1.44 *d* = -0.03	*t* = 1.16 *d* = 0.02
Guards food	*F* = 13.25, ω^2^ = 0.01***	*t* = 1.07 *d* = 0.02	*t* = -4.58 *d* = -0.10***	*t* = -5.03 *d* = -0.11***

Note:

* p < 0.05,

** p < 0.01,

*** p < 0.001. Due to multiple comparison inflation of Type I error, only effects of p < 0.001 should be considered notable.

F values are the test statistics of ANOVA. t values are the test statics of t tests.

### Feline results

#### Cat diets

One thousand, four hundred and eighteen cat owners responded, each describing a single cat. Of these 1,397 indicated the main diet their cat was fed. The three major dietary groups reported were conventional meat (1,178), raw meat (64) and vegan (127). In total 1,369 cats were jointly maintained on these three diets, with small numbers reportedly fed laboratory-grown meat diets (9), vegetarian diets (3), and insect-based diets (1). The remainder reported using mixtures (7) or were unsure (8).

**Fig 3:**
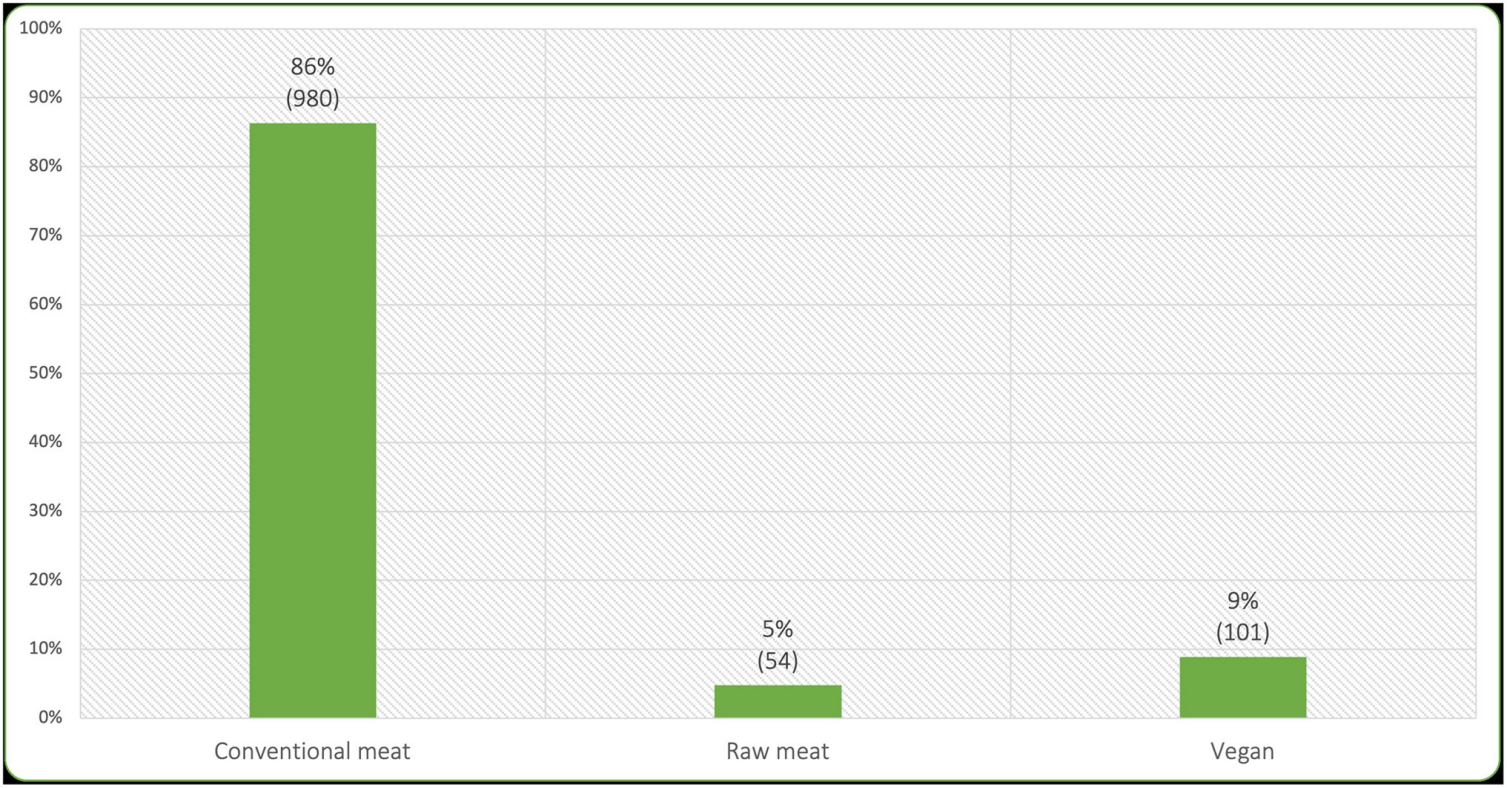
Diets of 1,135 sampled cats.

Owners were asked whether their cats normally competed with any others at mealtimes. After eliminating 234 ‘yes’, ‘unsure’ or ‘blank’ answers, 1,135 cats remained in the three main dietary groups who reportedly did not compete for food (Fig 3). These 1,135 cats comprised the sample subjected to further analysis. Food was available *ad libitum* for 37.2% (422) of these cats.

#### Sex/neuter status and age

**Fig 4:**
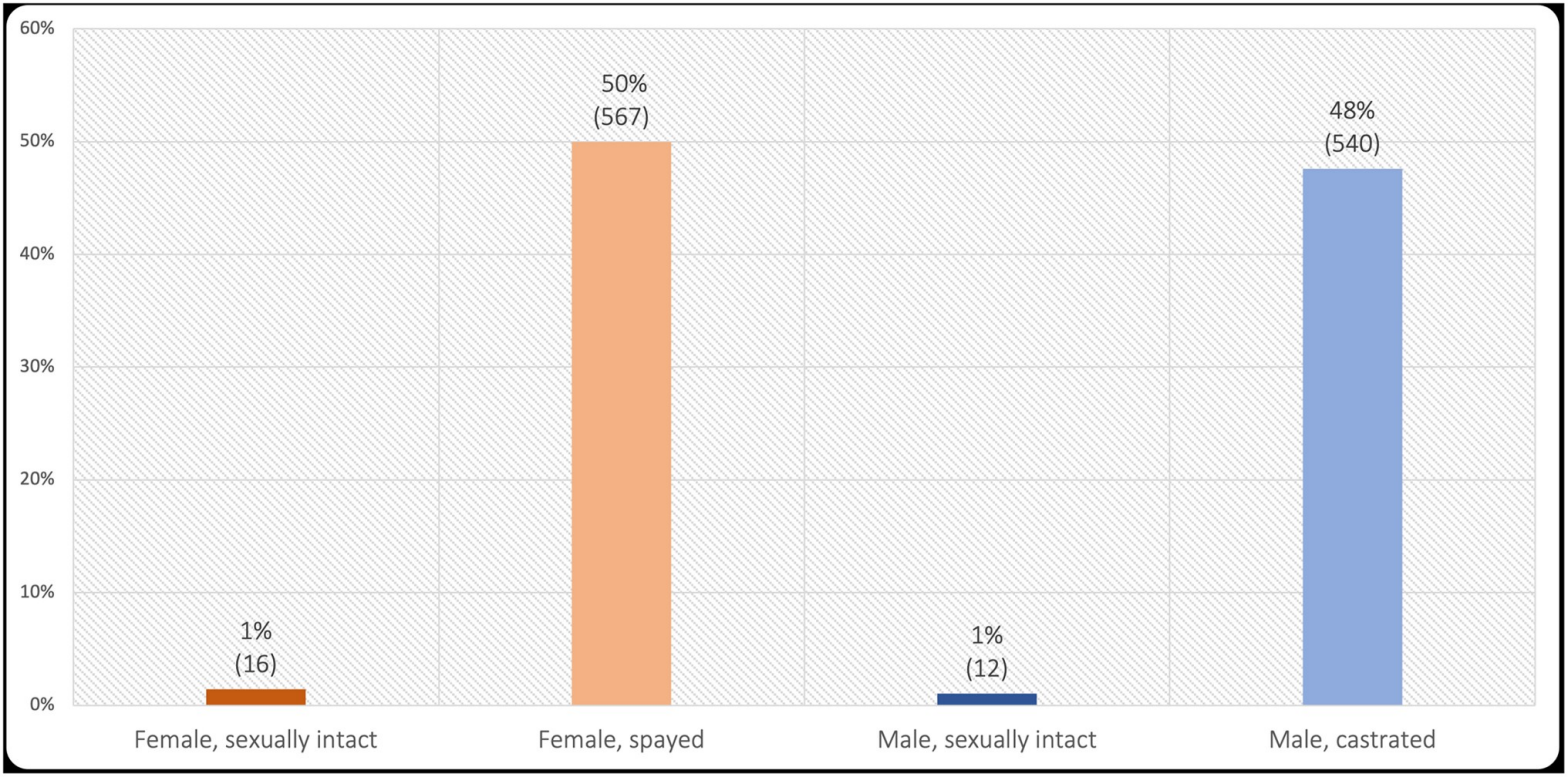
Sex/neuter status of 1,135 sampled cats.

The sex/neuter status of these 1,135 cats is provided in Fig 4. Neutered animals (those spayed or castrated) comprised 97% of the females, and 98% of the males. Much like the canine sample, was no notable variation in sex/neuter status with diet type (χ^2^(6) = 29.10, *p* < 0.001, Cramer’s *V* = 0.11). Similarly, the differences in palatability behaviours between sex/neuter groups were negligible (all *F* ≤ 4.21, all ω^2^ ≤ 0.01 with the largest being for tail flicking behaviour). Overall, there was no notable evidence that sex/neuter status affected diet type, or owner-reported behaviours.

Between the three main dietary groups studied, there was a significant, notably small difference, in cats’ mean ages (*F*(2, 1124) = 6.40, *p* = 0.002, ω^2^ = 0.01). Vegan cats were the youngest (*M* = 6.55, *SD* = 3.85) compared to those on conventional meat (*M* = 8.25, *SD* = 4.87, *t*(1124) = 3.85, *p* = 0.002, *d* = 0.36) diets. There was no significant difference between those cats fed raw meat diets (*M* = 7.34, *SD* = 4.35) and those fed conventional meat (*t*(1124) = 7.31, *p* = 0.525, *d* = 0.19) or vegan diets (*t*(1124) = 3.85, *p* = 0.983, *d* = 0.17). Age did not meaningfully relate to any of the owner-reported behaviours, with all correlations being negligible in size (all *r* ≤ |0.11|, with the largest being licking nose behaviour).

#### Feline palatability indicators

Of principle interest were behavioural reactions of the cats when provided with meals. As with the dog owners, cat owners were asked to report the extent to which they thought their cat displayed 15 behaviours (on a scale of Strongly Disagree (1) to Strongly Agree (5)). Results for the 15 feline palatability are provided in the S2 Appendix. Lack of reporting in some cases reduced total numbers.

#### Effect of activity level

As with the dogs, we also sought to determine the effects of activity level on cats’ behavioural responses to food. We similarly categorised the cats into more active (*n* = 589) and more sedentary (*n* = 546) activity levels, depending on whether they spent more or less than 60 minutes daily being active. There was no difference in activity level by diet (χ^2^(2) = 0.11, *p* = 0.947, Cramer’s *V* = 0.01). The food-oriented behaviours did not meaningfully differ by activity level (all *t* ≤ 2.43, *p* ≥ 0.015, *d* ≤ 0.15). In ANOVA all diet by activity level interactions on food-oriented behaviours were not significant (*F* ≤ 2.85, *p* ≥ 0.165, ω^2^ ≤ 0.00). This suggests that activity level of the cats did not impact their food-oriented behaviours.

#### Dimensions of behaviour

There was consistency in the ratings of the 15 food-oriented behaviours for the cats in the sample. This was demonstrated using McDonald’s ω for consistency, with a value of = 0.75. In this test alone, only the ‘leaving food uneaten’ behaviour was reverse scored, due to a first pass consistency analysis indicating that this item was inversely correlating with other items. This change meant that for the reliability analysis a high score was indicative of *less* frequently leaving food uneaten, in line with the other measures. For the rest of the analysis in this paper this behaviour is left forward scored.

Beyond overall consistency, we investigated the patterns of reporting food-oriented behaviour using factor analysis. Parallel analysis of the model fits of an oblimin exploratory factor analysis suggested that there were five overall factors that satisfactorily explained the reports of cats’ behaviour (RMSEA = 0.07 [0.06, 0.08], TLI = 0.86). Note that grooming behaviours did not adequately fit with any of these models. One factor summarised general licking behaviour by the cats, with the licking lips (0.76), nose (0.83), food (0.44) and bowl (0.48) behaviours all having similar patterns of responses. The approach behaviours grouped into another factor containing rapid approach to food (0.99), eating food quickly (0.51) and vocalising (0.46). The flicking/twitching behaviours grouped together with flicking of the ears (0.64) and the tail (0.71) receiving similar reports. Defensive behaviours were staying near the bowl (0.62) and guarding food (0.58). Eating quickly was also related to this factor (0.30). Cautious behaviours were summarised in a factor containing sniffing and investigating food (0.54), licking food (0.40), dropping food when eating (0.35), and leaving food uneaten (0.62).

The five discrete behavioural patterns identified were conservatively assessed as positive or negative palatability indicators, in this sample of 1,135 cats, based on knowledge of feline behaviour, and on previous studies of feline palatability indicators [44–47] (Table 4).

**Table 4:** Behavioural indicators of positive, negative or indeterminate palatability in 1,135 sampled cats.

**Positive (5)**	rapid approach to food, vocalisations, eating quickly, remaining near food bowl, guarding food
**Negative (4)**	lick food, sniff/investigate food, drop food, leave food uneaten
**Indeterminate (6)**	flick ears, flick tail, lick lips, lick nose, lick bowl, groom body

#### Tests of dietary difference

Table 5 reports on the distribution of reports of the cats’ food-oriented behaviour, with Table 6 reporting inferential tests of differences by diet. There were two notable patterns of effects. First, cats fed vegan diets were reported as licking their food less often than for those fed conventional meat (*d* = 0.56, see Table 2) and raw meat (*d* = 0.53. see Table 5) diets. Second, the cats fed conventional meat diets were more likely to leave their food unfinished than those fed raw meat (*d* = 0.59) and to a lesser extent vegan diets (*d* = 0.38).

**Table 5:** Descriptive statistics of the owner reported food-oriented behaviours, with range Strongly Disagree (1) to Strongly Agree (5).

Factor	Variable	Diet		
		Conventional Meat	Raw Meat	Vegan
		Mean (SD)	Mean (SD)	Mean (SD)
Approach	Rapid Approach	3.86 (1.11)	4.11 (0.97)	4.02 (0.92)
Approach	Vocalisations	3.85 (1.29)	4.11 (1.17)	3.82 (1.16)
Approach/Defensive	Eating Quickly	3.25 (1.22)	3.42 (1.18)	3.32 (1.09)
Defensive	Stays Near Bowl	2.68 (1.21)	2.75 (1.26)	2.71 (1.12)
Defensive	Guards Food	1.88 (0.95)	1.88 (0.96)	1.92 (0.87)
Flicking	Flick Ears	2.32 (1.00)	2.55 (1.19)	2.02 (0.91)
Flicking	Flick Tail	2.68 (1.15)	2.90 (1.20)	2.35 (1.05)
Licking	Licking Lips	3.18 (1.18)	3.21 (1.24)	3.27 (1.22)
Licking	Licking Nose	2.78 (1.11)	3.13 (1.16)	2.94 (1.09)
Licking/Cautious	Licking Food	3.65 (1.18)	3.62 (1.19)	2.99 (1.18)
Licking	Licking Bowl	3.03 (1.30)	3.37 (1.25)	2.74 (1.11)
Cautious	Sniff/Investigate	3.70 (1.09)	3.68 (1.14)	3.36 (1.10)
Cautious	Drop Food	2.76 (1.15)	2.77 (1.20)	2.48 (0.97)
Cautious	Leaving Food Uneaten	3.49 (1.19)	2.79 (1.23)	3.04 (1.14)
Unclassified	Grooming	3.12 (1.29)	3.37 (1.37)	3.12 (1.22)

**Table 6:** Comparison between diet groups for the owner-reported food-oriented behaviour.

Variable	Differences between all three diets	Conventional vs Raw	Conventional vs Vegan	Raw vs Vegan
Rapid Approach	*F* = 2.25, ω^2^ = 0.00	*t* = -1.67 *d* = -0.24	*t* = -1.42 d = -0.15	*t* = 0.50 *d* = 0.09
Vocalisations	*F* = 1.13, ω^2^ = 0.00	*t* = -1.47 *d* = -0.21	*t* = 0.21 d = 0.02	*t* = 1.35 *d* = 0.23
Eating Quickly	*F* = 0.61, ω^2^ = 0.00	*t* = -0.99 *d* = -0.14	*t* = -0.55 d = -0.06	*t* = 0.48 *d* = 0.08
Stays Near Bowl	*F* = 0.10, ω^2^ = 0.00	*t* = -0.38 *d* = -0.05	*t* = -0.27 d = -0.03	*t* = 0.15 *d* = 0.03
Guards Food	*F* = 0.09, ω^2^ = 0.00	*t* = -0.04 *d* = -0.01	*t* = -0.43 d = -0.05	*t* = 0.24 *d* = -0.04
Flick Ears	*F* = 4.47, ω^2^ = 0.01	*t* = -1.41 *d* = -0.22	*t* = 2.54 d = 0.30	*t* = 2.73 *d* = 0.52
Flick Tail	*F* = 4.42, ω^2^ = 0.01	*t* = -1.32 *d* = -0.19	*t* = 2.57 d = 0.29	*t* = 2.72 *d* = 0.49
Licking Lips	*F* = 0.26, ω^2^ = 0.00	*t* = -0.18 *d* = -0.03	*t* = -0.71 d = -0.08	*t* = -0.29 *d* = -0.05
Licking Nose	*F* = 2.93, ω^2^ = 0.00	*t* = -2.12 *d* = -0.32	*t* = -1.30 d = -0.15	*t* = 0.92 *d* = 0.17
Licking Food	*F* = 12.90, ω^2^ = 0.02***	*t* = 0.19 *d* = 0.03	*t* = 5.08 d = 0.56***	*t* = 3.05 *d* = 0.53***
Licking Bowl	*F* = 4.26, ω^2^ = 0.01	*t* = -1.82 *d* = -0.26	*t* = 2.15 d = 0.23	*t* = 2.84 *d* = 0.49
Sniff/Investigate	*F* = 4.39, ω^2^ = 0.01	*t* = 0.17 *d* = 0.02	*t* = 2.96 d = 0.31	*t* = 1.70 *d* = 0.29
Drop Food	*F* = 2.65, ω^2^ = 0.00	*t* = -0.04 *d* = -0.01	*t* = 2.30 d = 0.25	*t* = 1.45 *d* = 0.25
Leaving Food Uneaten	*F* = 14.20, ω^2^ = 0.02***	*t* = 4.15 *d* = 0.59***	*t* = 3.63 *d* = 0.38***	*t* = -1.24 *d* = 0.21
Grooming	*F* = 0.96, ω^2^ = 0.00	*t* = -1.39 *d* = -0.20	*t* = -0.02 *d* = 0.00	*t* = 1.13 *d* = 0.20

Note:

* *p* < 0.05,

** *p* < 0.01,

*** *p* < 0.001. Due to multiple comparison inflation of Type I error, only effects of *p* < 0.001 should be considered notable.

F values are the test statistics of ANOVA. t values are the test statics of t tests.

Overall, the evidence here suggests that the diets made little difference to the food-orientated behaviour of the cats in this sample, except perhaps that cats on vegan diets lick their food less often, and cats on conventional diets leave more food. It should be noted that the comparison groups in these tests are particularly small, and inferences should be treated with caution.

## Discussion

### Importance of palatability to owners

Palatability was one of the factors considered most important by owners, when choosing pet diets. In order of priority, the three factors considered most important to consumers when choosing pet diets, were health and nutrition, diet quality, and palatability. The latter was cited as important by 69.5% of 3,976 respondents who played at least some role in choosing diets.

The importance of palatability has also been identified in other studies, such as that of Schleicher and colleagues [59], in their survey of 2,181 surveyed respondents. “Pet preference” was the sixth most important determinant of diet choice. The slightly lower ranking compared to our results, may result from more fine-grained respondent options, with more choices. Our respondents could choose among 12 factors (including ‘other’ but excluding ‘none’), whereas those of Schleicher and colleagues could choose among 25.

Among our 3,408 respondents currently feeding conventional or raw meat diets, 46.5% stated they would consider an alternative diet, if it offered their desired attributes and standards. In order of priority, these were confidence about pet health, and confidence about nutritional soundness (both of equal importance), good quality, and palatability, with the latter cited as important by 70.5% of respondents.

Dodd and colleagues [76] similarly surveyed pet owners. They reported that 34.6% (1,083/3,130) of pet owners who did not already feed a plant-based diet to their pet, were interested in doing so. The slight decline compared to our 46.5% willing to consider alternative diets, may be attributable to (i) the ‘alternative diets’ offered as choices to our respondents, being broader than the plant-based diets offered as choices by and colleagues, although at the present time, most alternative diets are indeed plant-based, and (ii) demographic differences. Our respondents were overwhelmingly from the UK and Europe, whereas those surveyed by Dodd and colleagues were primarily North American.

### Behavioural indicators of palatability

In analysing the observations of 2,308 dogs and 1,135 cats, our study included many more participants than is normal for palatability studies [58]. The reported observations of these dogs and cats revealed discrete behavioural patterns (four in dogs, five in cats) which highlighted similarities between food-oriented behaviours the owners reported here. We used these to conservatively interpret these behaviours as positive, negative, or indeterminate indicators of palatability (Tables 1 and 4). In the case of dogs, our findings were generally consistent with those of previous investigators [40–43].

However, some of the indicators of feline palatability that we identified confirmed prior studies, whilst others did not. We identified as positive indicators, rapid approach to food and eating quickly. Yet, these were not identified as positive indicators by Becques and colleagues [46]. Our analysis identified sniffing food as a negative indicator. This confirmed the observations of Van den Bos and colleagues [44], and Becques and colleagues [46].

Licking of lips, nose, and bowl followed similar patterns of responses. These also varied consistently with licking of food, and the latter varied consistently with several other behaviours assessed as negative indicators. This provisionally suggested that licking lips, nose, and bowl might also be negative indicators. However, these provisional findings were inconsistent with those of several previous studies. Hence, we felt unable to make a judgement, as to whether these behaviours indicated positive or negative palatability. Previously, bowl and lip licking were both observed to indicate good palatability by Van den Bos and colleagues [44], and lip licking was also concluded by Savolainen and colleagues [45] to be a positive sign. Nose licking was assessed as a positive indicator by Hanson and colleagues [47], but as a negative indicator by Van den Bos and colleagues [44], and Savolainen and colleagues [45].

Flicking of ears and the tail had similar patterns of responses, but we did not feel we had enough information to be able to assess these as positive or negative indicators of palatability. Savolainen and colleagues [45], however, labelled these as negative indicators. Body grooming did not vary consistently with any of the other responses, and we similarly assessed it as ‘Indeterminate’, although Savolainen and colleagues [45], also labelled this as a negative indicator. Leaving food uneaten varied consistently with three other indicators assessed as negative indicators of palatability, and so was similarly assessed as a negative indicator. Finally, as noted by Tobie and colleagues [6], some feline indicators, such as sniffing and licking behaviour, seem not to vary with palatability, when dry food is offered, as occurs when wet food is offered.

### Palatability variation with diet type

This study primarily sought to investigate whether behaviour indicators of palatability varied with main diet type. We did find significant but small effects of increased reports of appetitive behaviour by dogs on a raw meat diet, as opposed to a conventional diet, and these were consistent. Very occasional differences between vegan diets, and both the conventional and raw meat diets, were not consistent. Apart from these, the behavioural indicators studied did not generally vary when dogs or cats were fed conventional meat, raw meat and vegan diets.

### Potentially confounding factors

Among dogs, appetitive behaviours were minimally influenced by activity level (i.e., exercising more or less than one hour daily). Dogs on vegan diets were slightly more likely to be sedentary than those on conventional meat or raw meat diets. Only jumping and vocalisation behaviours were significantly different between the active and sedentary groups, and these differences were notably small, suggesting a limited effect of activity level on food-oriented behaviours. For example, the owners in the active group were on average ‘neither agreeing nor disagreeing’ (a scale response of 3) with the jumping in response to food question (M = 2.62, SD = 1.44), as were those in the sedentary group (M = 2.86, SD = 1.47). For cats, the different diets were not associated with statistically significant differences in activity levels.

The feeding strategy of cats would naturally involve the consumption of several small prey animals daily [16]. This might be expected to affect the behavioural sign of ‘leaving food uneaten’, especially for cats fed *ad libitum*. However, only 37.2% of the cats we studied were fed *ad libitum*, and ‘leaving food uneaten’ did vary consistently with three other indicators assessed as negative indicators of palatability. Hence, we felt this behavioural sign was a valid indicator of negative palatability. This behavioural sign varied between dietary groups, with cats fed conventional meat diets more commonly leaving food uneaten, than cats fed raw meat or vegan diets.

Our results were unaffected by age variations between dietary groups. Small, significant differences in mean ages did exist. For dogs, mean ages in years ranged from 5.44 (raw meat), 6.35 (conventional meat) to 7.26 (vegan). For cats mean ages ranged from 6.55 (vegan), raw meat (7.34) to conventional meat (8.25). However, age did not meaningfully relate to any of the owner-reported behaviours, with all correlations being negligible in size, for both dogs and cats. Our results were similarly unaffected by sex/neuter variations between dietary groups. For both dogs and cats, there was no notable evidence that sex/neuter status affected diet type, or owner-reported behaviours.

### Implications

Our results concur with some of the existing, limited studies in this field. After finding minimal evidence of palatability variation between plant- and meat-based diets, in their study of eight beagles, Callon and colleagues [41] concluded that dogs lack innate preferences for animal- or vegetable-based diets similar to those found in commercial formulas. They concluded that any apparent differences in interest levels may be due to other factors, such as level of satiety, or specific ingredients or processing techniques employed to enhance palatability.

When considered overall, the results of our study, and others in this field, do not support the position that vegan diets have poorer palatability, or compromise pet welfare, when compared to conventional meat or raw meat diets; provided that other determinants of welfare, such as nutritional soundness, are adequately addressed. The 2,308 dogs and 1,135 cats in our study generally displayed a variety of signs indicative of positive anticipation and enjoyment of their meals, and of the value they accord to these, to the extent that these subjective affective states and values may be inferred from external behaviour.

Good welfare requires, among other things, the provision of diets that are nutritious, the opportunity to exhibit natural behaviour, and a positive mental state. The nutritional suitability of various dietary options are outside the scope of this article, but are addressed elsewhere (e.g. [23–30, 77, 78], and in forthcoming studies by the lead author and colleagues). However, the different dietary options explored within this study did not generally appear to result in significant behavioural differences associated with meals, nor in mental states, at least to the extent that these may be inferred from behaviour.

Guardians concerned with the welfare of their animals might better focus their attention on other factors related to feeding. Dogs, for example, have been domesticated at least 14,000 years, and possibly as long as 40,000 years [79]. They evolved in close social contact with humans for much of that period, successfully scavenging leftovers from human meals [32]. Accordingly, feeding them concurrently with their human family may provide beneficial social enrichment, provided this does not create competition or resource-guarding between animals. Similarly, facilitating their natural appetitive, problem solving behaviours may benefit their welfare. Dogs are ‘contra-freeloaders’–meaning that they find it more rewarding to work for their food, than to obtain it freely [80]. Allowing dogs to solve problems and work for their food can be as simple as scattering kibble across lawns or floors, or can involve home-made or commercial activity feeders [81]. Schipper and colleagues [82] demonstrated that a relatively simple feeding enrichment toy such as a rubber toy stuffed with dog treats can stimulate appetitive behaviours, increase activity level and behavioural variety, and lower barking frequencies—all of which can be beneficial. Similarly, Zoran and Buffington [16] noted that use of puzzle feeders and hiding food in different locations can encourage cats to exhibit more active (and natural) behaviour, and can enhance muscular activity and neurocognitive function, which can benefit wellbeing [83].

Physical characteristics may also be more important than main ingredient type. Sagols and colleagues [41] demonstrated that changing kibble cross-sectional shape from round to cross, resulted in more chewing behaviour, slower ingestion speed and a significant decrease in food-seeking behaviour, reducing voluntary food intake. Incidentally, this is particularly beneficial when seeking to manage excessive body weight. Obesity is one of the most common health disorders in dogs and cats [84]. In 2018, 59.5% of cats and 55.8% of dogs in the US were estimated as overweight or obese [85]. Obesity was also the third most prevalent specific health disorder (after periodontal disease—first, and otitis externa—second) among 22,333 dogs randomly sampled from a population of 905,543 dogs under veterinary care at 886 UK clinics in 2016. Obesity was present in 7.07% of these dogs (95% CI: 6.74–7.42) [86].

### Study limitations

Unlike panels of trained and experienced animal testers, with tests conducted under standardised conditions, family owned pets may have greater variability of potentially confounding factors, such as age, sex, breed and dietary history. Pets are also likely to be less experienced with a variety of test diets. Owner assessments of behaviour, conducted by numerous, untrained individuals, are also less likely to be reliable, than those of trained and consistent human assessors. To accommodate such increased variation, Tobie and colleagues [6] recommended that palatability tests utilise large groups (ideally, approximately 100 animals), compared to the minimum of 30 more commonly used in industry or research testing, to achieve statistical robustness. All groups examined in this study achieved such larger numbers, other than cats fed raw meat diets (n = 54). Accordingly, caution should be exercised about the results in this group of animals.

### Suggestions for further research

The positive and negative canine indicators of palatability, identified after analysing the behavioural responses of these 2,308 dogs, were relatively unambiguous, and generally confirmed prior studies. However, it was less clear whether some of the feline behaviours observed indicated positive or negative palatability. Our analysis did reveal which behaviours were most often reported together. Some of our feline findings concurred with, while others contradicted, previous literature, and some other studies also contradicted one another. As noted by Pires and colleagues [58], sample sizes in feline palatability studies normally vary from nine to 60. Although we analysed observations of 1,135 cats, an even larger feline study might help resolve some of this uncertainty, as might studies conducted under more standardised conditions, using more trained and experienced animal testers or observers.

Additionally, alliesthesia (the extent to which perceived pleasure or displeasure of stimuli may be affected by physiological status, and the need to maintain homeostasis), could theoretically affect preferences for specific nutrients and flavours, as well as overall food intake [87]. And indeed, preference for specific nutrient profiles have been demonstrated by both dogs and cats [88, 89]. Factors affecting physiological status, with the potential to exert such effects, include breed, age, sex, neutering status, body condition and weight, health status, exercise levels, seasonality or social factors with the potential to alter ambient temperature or stress levels, with resultant effects on metabolic requirements. Little information has yet been published investigating the effects of most of these factors on palatability [50]. Further research could aim to do so, but might require limiting to groups of specific interest, rather than cats and dogs in general as in this study, to ensure sample sizes are sufficient to allow statistically significant results.

## Conclusions

The global pet population and pet food markets are substantial. Consumer suspicion of industrially-produced pet foods, along with increasing concerns about the environmental sustainability of conventional pet foods, and the perceived health benefits of alternative diets, are driving considerable development of alternatives, such as vegan pet foods. Some have asserted that such diets may be less palatable [37], or that they may compromise animal welfare [17, 18]. This is an important consideration for consumers, and hence ultimately, the success of such diets. Of 3,976 respondents to our survey who played some role in pet diet decision-making, palatability was the third most important among 12 factors cited as important when choosing pet diets. Among these respondents, 3,408 owners were feeding conventional or raw meat diets. Just under half of these stated they would realistically consider alternative diets. Palatability was the fourth most important among 14 attributes these respondents desired, within alternative diets.

Our study of the reported behavioural responses of dogs and cats to their meals, had sufficient numbers maintained on conventional, raw meat, and vegan diets, to draw some conclusions about the general palatability of vegan pet foods, in comparison to meat-based conventional and raw diets. For 2,308 dogs, consideration of 10 behavioural indicators associated with meals, reliably indicated small effects of increased reports of appetitive behaviour by dogs on a raw meat diet, as opposed to a conventional diet. There were few significant differences between vegan diets and either the conventional or raw meat diets. For 1,135 cats, consideration of 15 behavioural indicators also indicated that diet made little difference to the food-oriented behaviour of the cats studied, except perhaps that cats on vegan diets lick their food less often, and cats on conventional diets leave more food. However, the feline comparison groups (particularly, raw meat *n* = 54) were relatively small, so inference of differences should be treated with caution.

Although palatability is important, animal welfare also depends on a range of other factors. However, the results from our study, which concur with limited existing studies in this field, do not support views that vegan pet food may have reduced palatability, and thus compromise the welfare of dogs or cats in this manner.

## Supporting information

S1 Appendix Canine palatability indicators (10). (PDF)

S2 Appendix Feline palatability indicators (15). (PDF)
