# Correction: Vegan versus meat-based pet foods: Owner-reported palatability behaviours and implications for canine and feline welfare

**DOI:** 10.1371/journal.pone.0260198

**Published:** 2021-11-12

**Authors:** Andrew Knight, Liam Satchell

There are errors in the first and second sentences of the fourth paragraph in the “Potentially confounding factors” subsection of the Methodology. The authors apologize for the errors. The correct sentences are: Climate seasonality may also affect appetite. Our respondents were not geographically restricted, and we expected animals could experience a range of climatic conditions. Serisier and colleagues [64] found that cats consume more in winter. Similarly, Alegría-Morán and colleagues [51] found that feline food intake increased, especially in females, in winter.

## References

[1] Knight A, Satchell L (2021) Vegan versus meat-based pet foods: Owner-reported palatability behaviours and implications for canine and feline welfare. PLoS ONE 16(6): e0253292. 10.1371/journal.pone.0253292 34133456 PMC8208530

