# Supplementary material for: Vegan versus meat-based pet foods: Owner-reported palatability behaviours and implications for canine and feline welfare
Source: PLoS One. 2021 Jun 16;16(6):e0253292. doi: 10.1371/journal.pone.0253292 (PMC8208530; doi:10.1371/journal.pone.0253292)
Supplement: S1 Appendix — (PDF) [file pone.0253292.s001.pdf]

S1 Appendix – Canine palatability indicators (10)

1. Eats Quickly
2. Approach
3. Wag tail
4. Sniffs Food
5. Jump
6. Vocalise
7. Salivate
8. Licks lips
9. Stays near bowl
10. Guards food

Nb: Lack of reporting in some cases reduced total numbers, in some following Tables and Figures.

1. Ate quickly

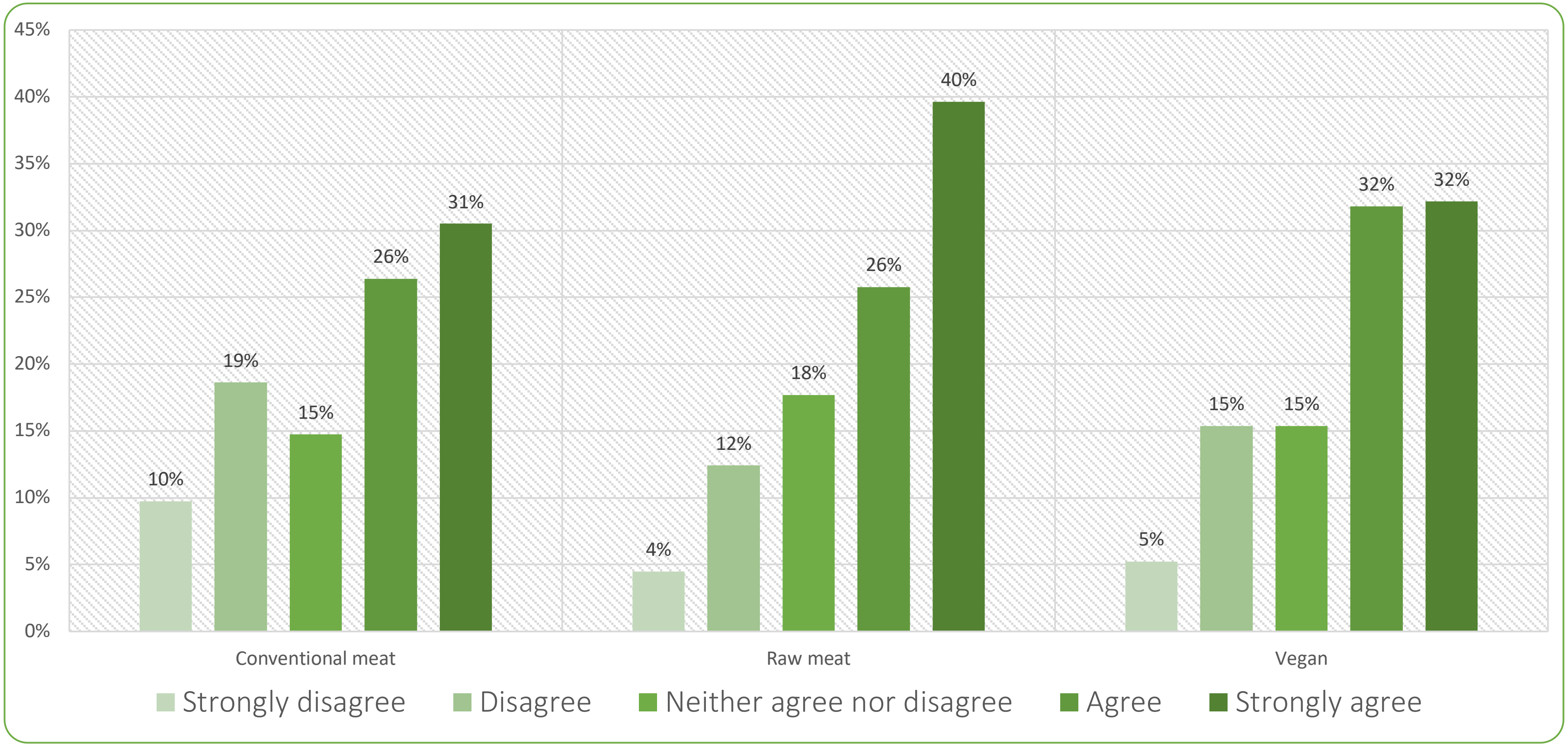

Figure A1. Percentage of dogs who ate quickly.

Table A1. Numbers of dogs who ate quickly.

Agreement level	Conventional meat	Raw meat	Vegan	Total
Strongly disagree	123	34	15	172
Disagree	235	94	44	373
Neither agree nor disagree	186	134	44	364
Agree	333	195	91	619
Strongly agree	385	300	92	777
Total	1262	757	286	2305

2. Approached meals rapidly

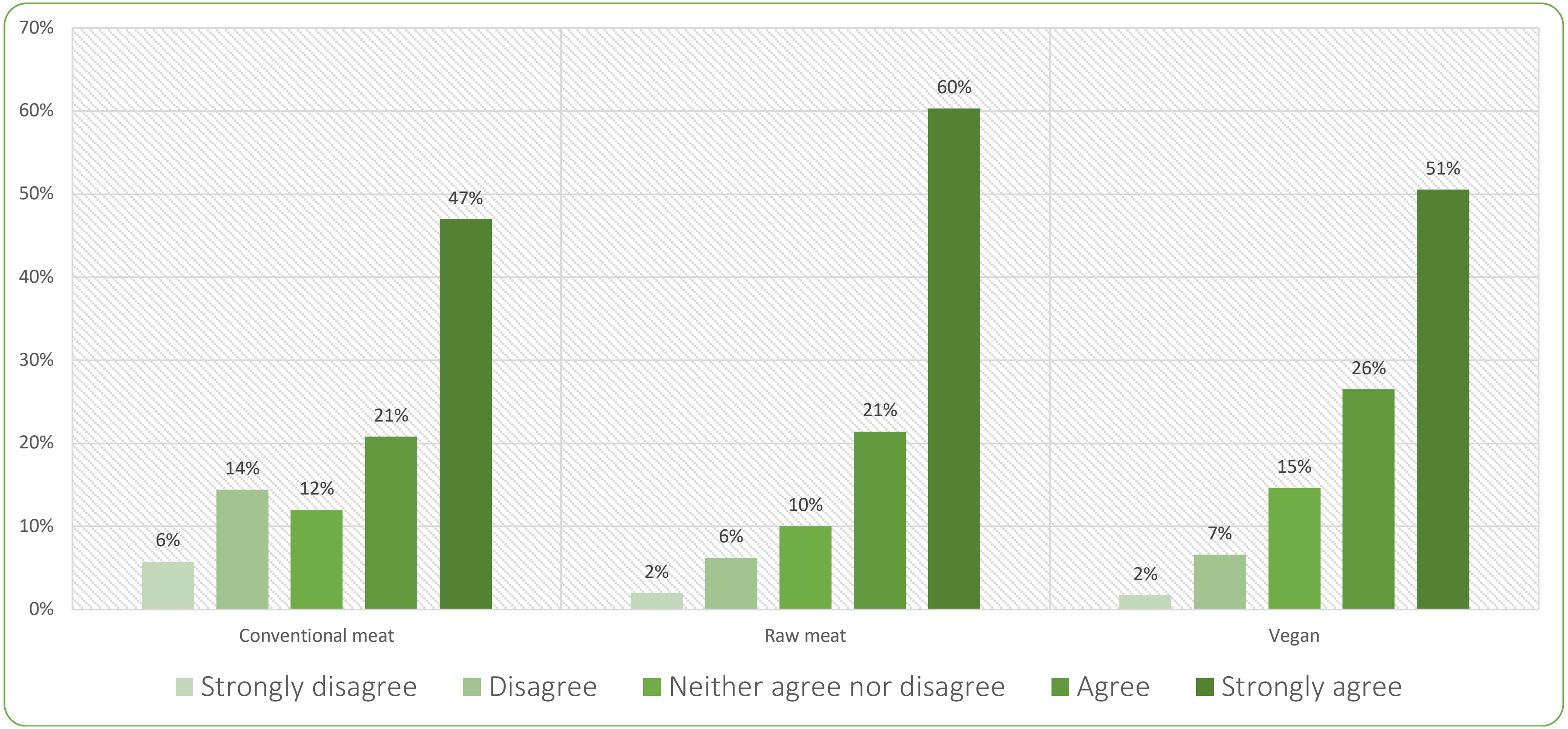

Figure A2. Percentage of dogs who approached meals rapidly.

Table A2. Numbers of dogs who approached meals rapidly.

Agreement level	Conventional meat	Raw meat	Vegan	Total
Strongly disagree	73	15	5	93
Disagree	182	47	19	248
Neither agree nor disagree	151	76	42	269
Agree	262	162	76	500
Strongly agree	592	456	145	1193
Total	1260	756	287	2303

3. Wagged tail

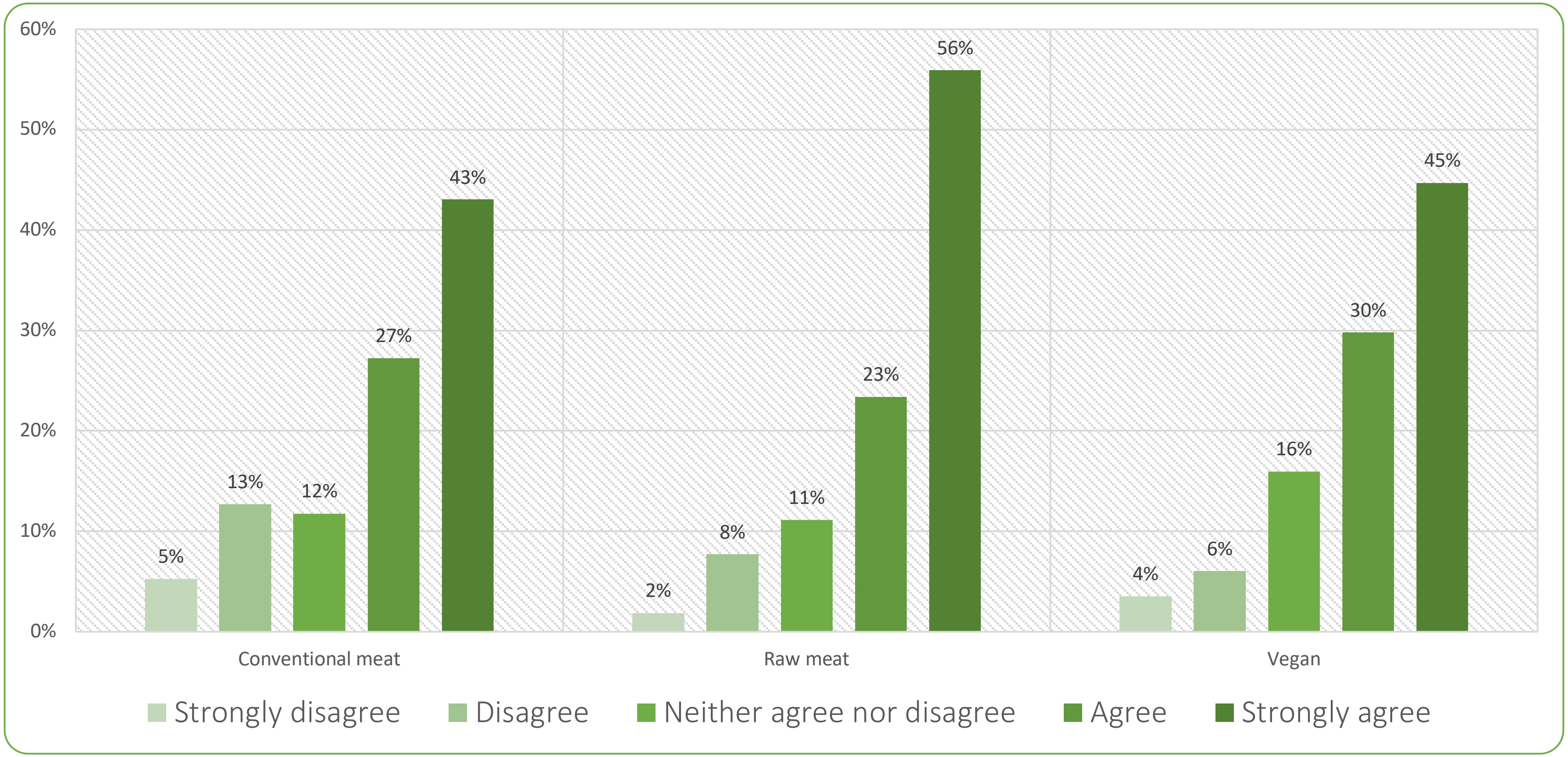

Figure A3. Percentage of dogs who wagged their tails.

Table A3. Numbers of dogs who wagged their tails.

Agreement level	Conventional meat	Raw meat	Vegan	Total
Strongly disagree	66	14	10	90
Disagree	159	58	17	234
Neither agree nor disagree	147	84	45	276
Agree	341	176	84	601
Strongly agree	539	421	126	1086
Total	1252	753	282	2287

4. Sniffed or investigated food

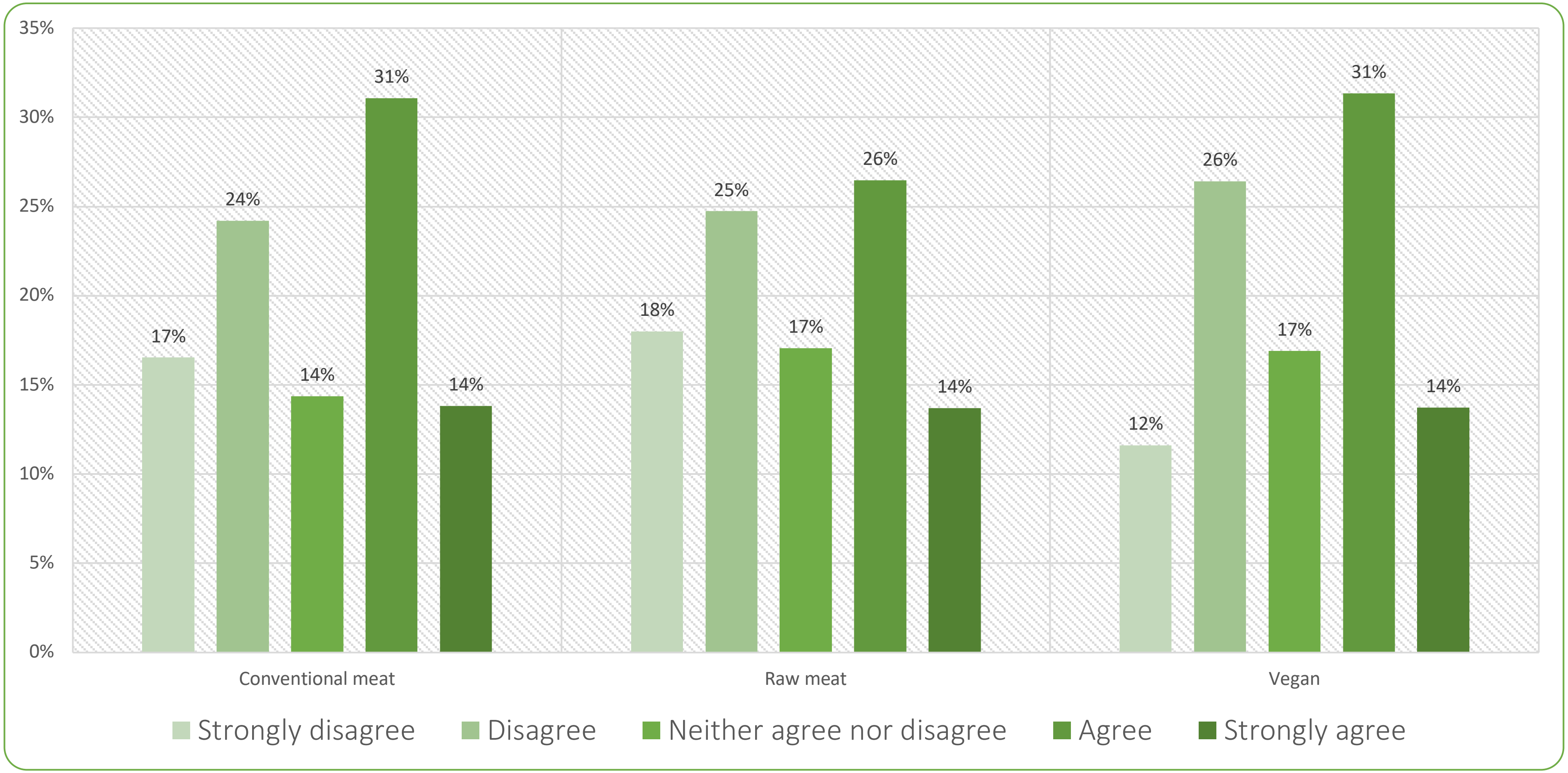

Figure A4. Percentage of dogs who sniffed or investigated food.

Table A4. Numbers of dogs who sniffed or investigated food.

Agreement level	Conventional meat	Raw meat	Vegan	Total
Strongly disagree	207	134	33	374
Disagree	303	184	75	562
Neither agree nor disagree	180	127	48	355
Agree	389	197	89	675
Strongly agree	173	102	39	314
Total	1252	744	284	2280

5. Jumped

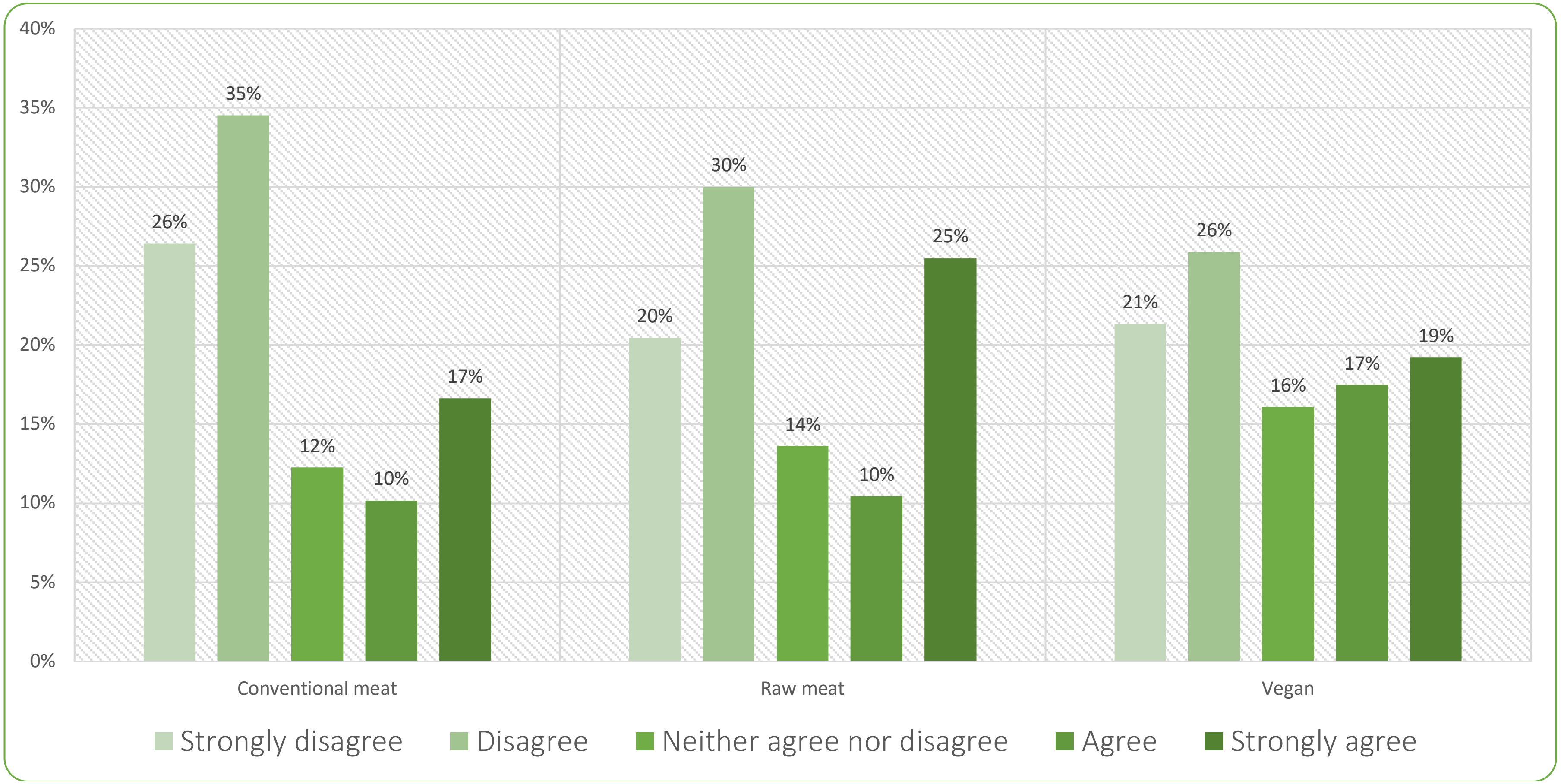

Figure A5. Percentage of dogs who jumped.

Table A5. Numbers of dogs who jumped.

Agreement level	Conventional meat	Raw meat	Vegan	Total
Strongly disagree	332	155	61	548
Disagree	434	227	74	735
Neither agree nor disagree	154	103	46	303
Agree	128	79	50	257
Strongly agree	209	193	55	457
Total	1257	757	286	2300

6. Barked or vocalised

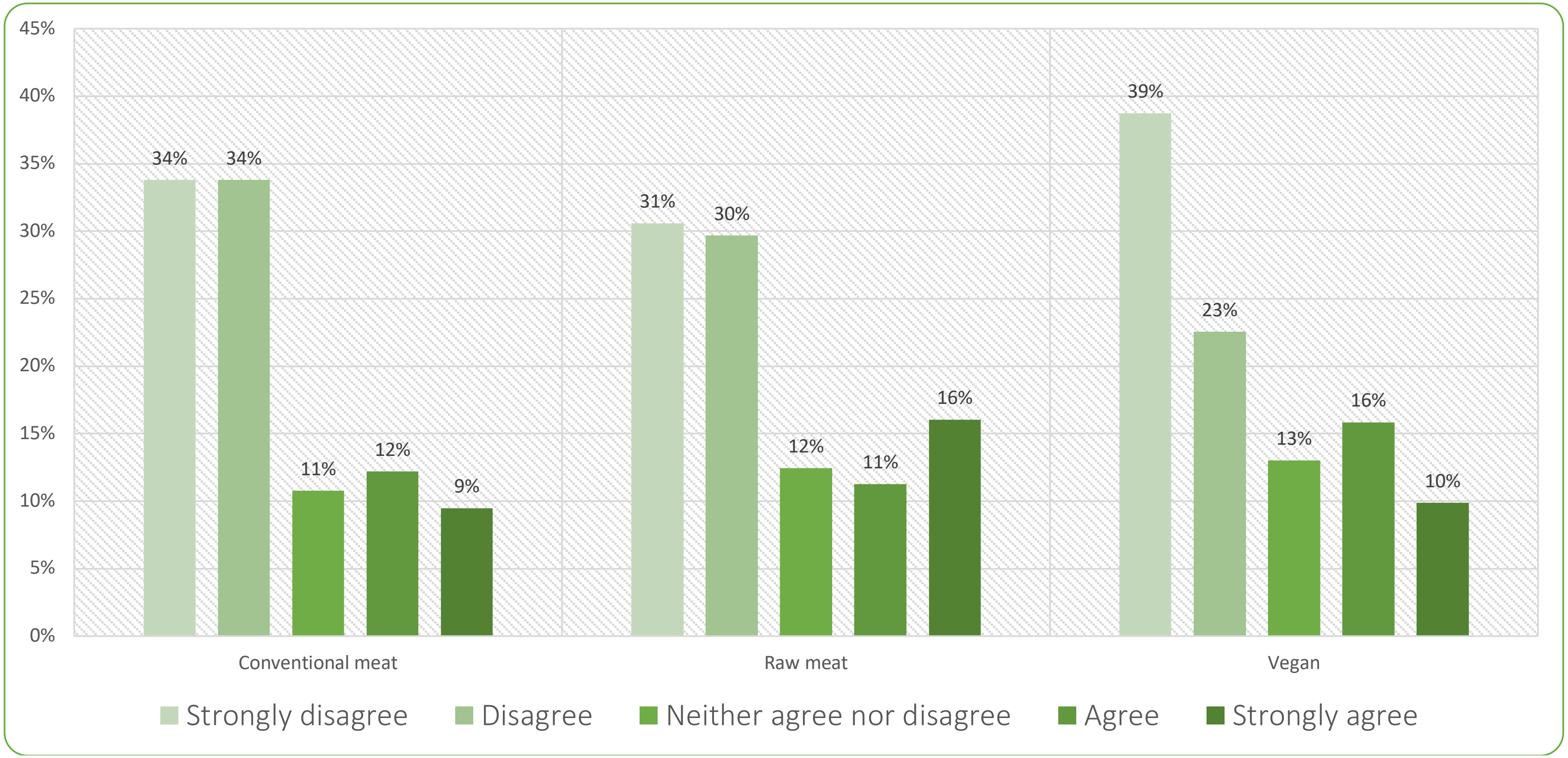

Figure A6. Percentage of dogs who barked or vocalised.

Table A6. Numbers of dogs who barked or vocalised.

Agreement level	Conventional meat	Raw meat	Vegan	Total
Strongly disagree	424	231	110	765
Disagree	424	224	64	712
Neither agree nor disagree	135	94	37	266
Agree	153	85	45	283
Strongly agree	119	121	28	268
Total	1255	755	284	2294

7. Obviously salivated

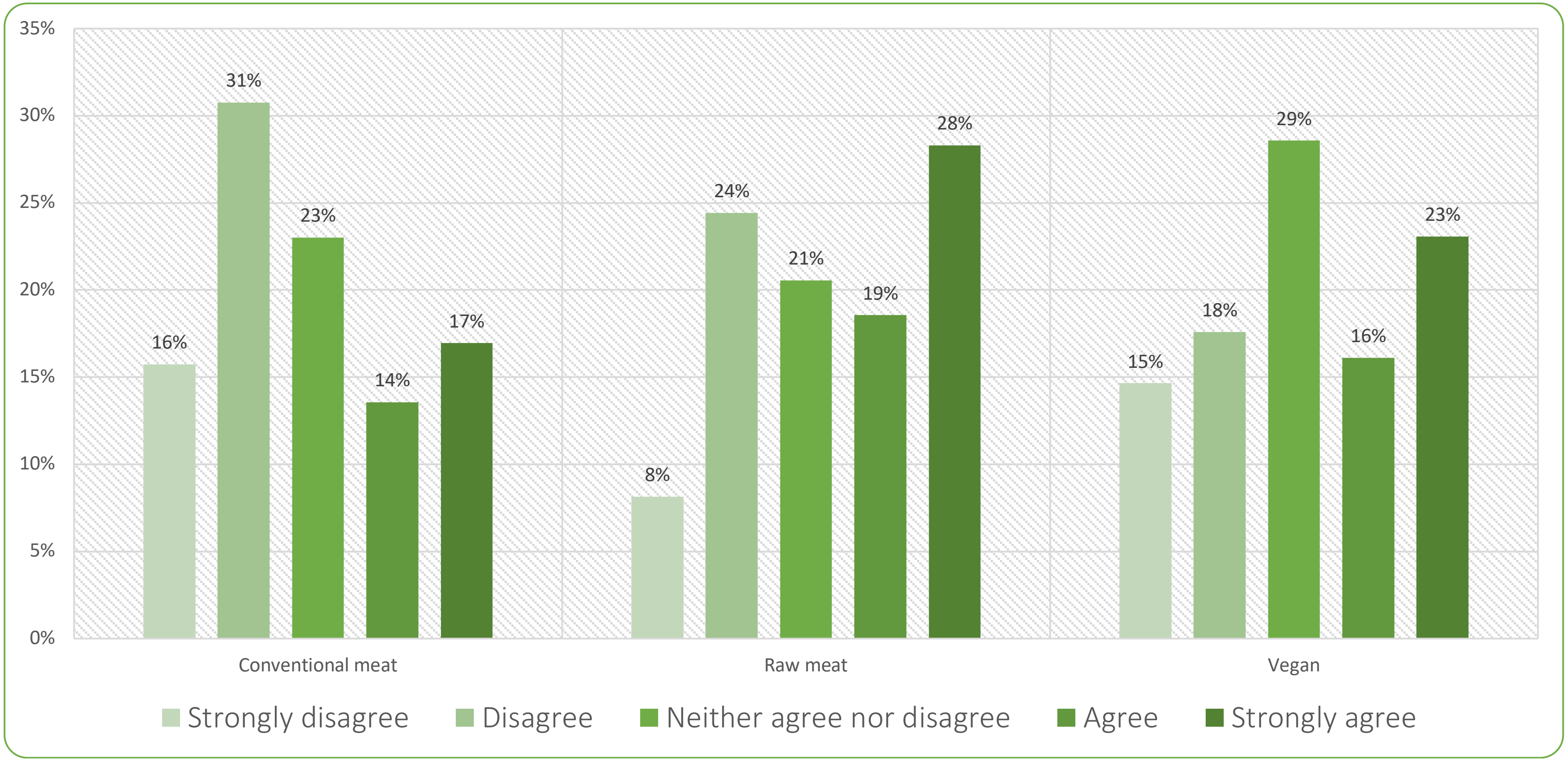

Figure A7. Percentage of dogs who obviously salivated.

Table A7. Numbers of dogs who obviously salivated.

Agreement level	Conventional meat	Raw meat	Vegan	Total
Strongly disagree	195	61	40	296
Disagree	381	183	48	612
Neither agree nor disagree	285	154	78	517
Agree	168	139	44	351
Strongly agree	210	212	63	485
Total	1239	749	273	2261

8. Licked lips

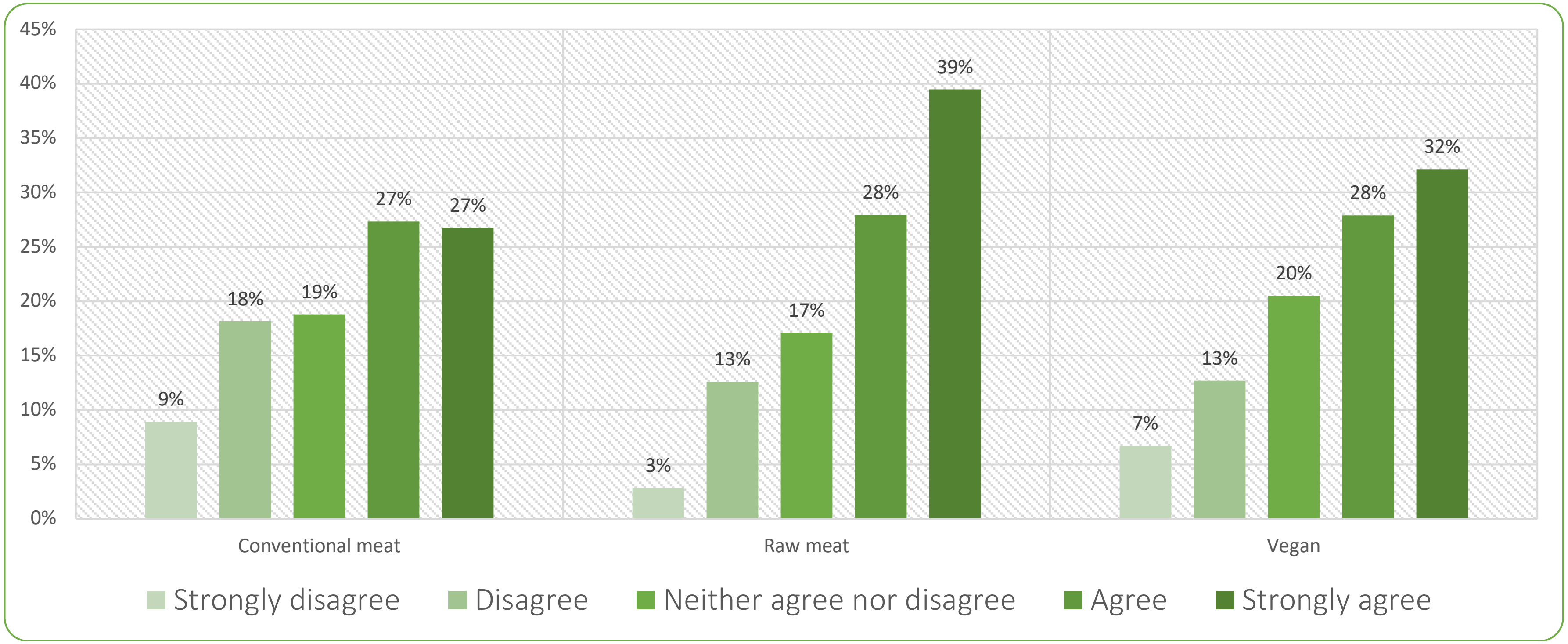

Figure A8. Percentage of dogs who licked their lips.

Table A8. Numbers of dogs who licked their lips.

Agreement level	Conventional meat	Raw meat	Vegan	Total
Strongly disagree	109	21	19	149
Disagree	222	93	36	351
Neither agree nor disagree	230	126	58	414
Agree	334	206	79	619
Strongly agree	327	291	91	709
Total	1222	737	283	2242

9. Remained near food bowl

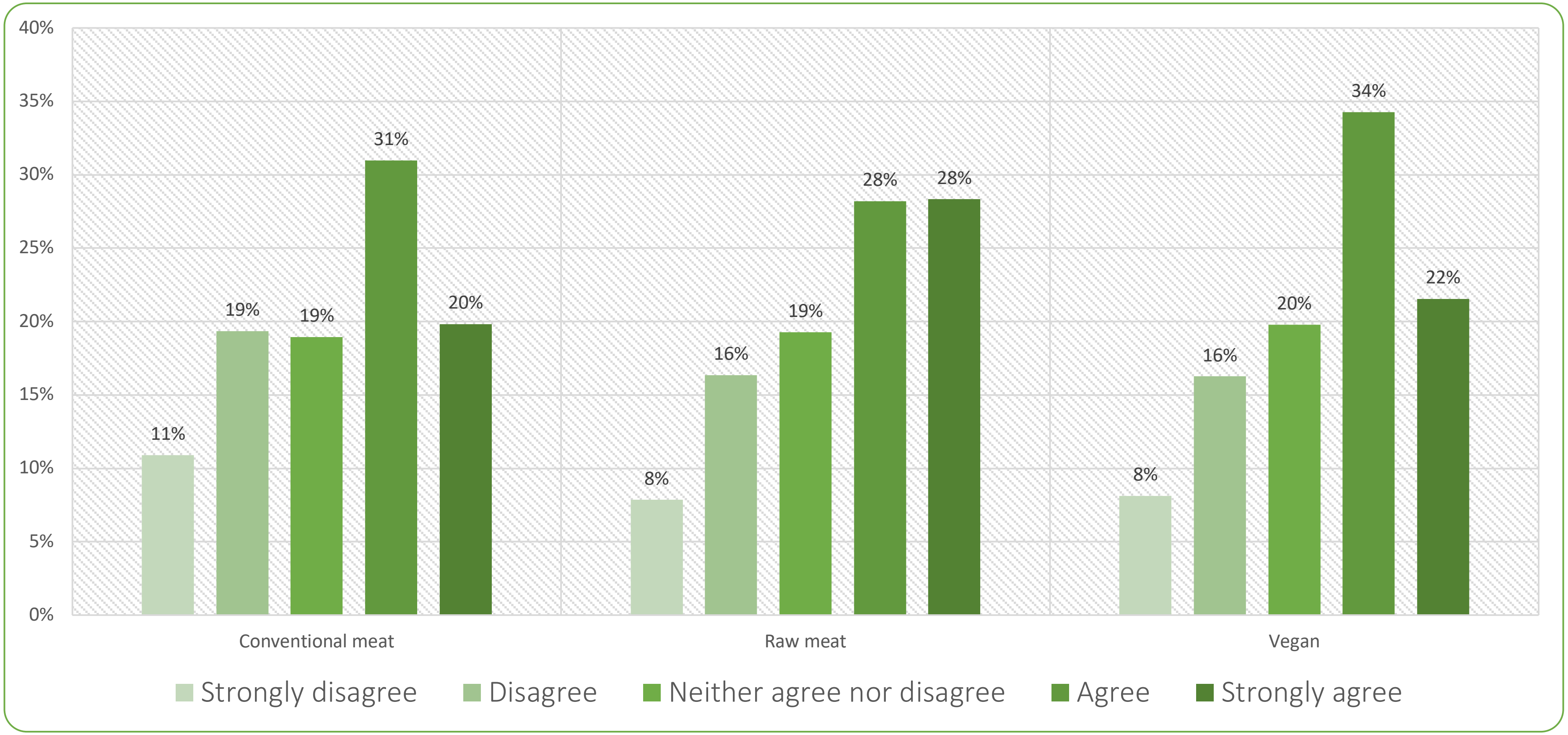

Figure A9. Percentage of dogs who remained near their food bowl.

Table A9. Numbers of dogs who remained near their food bowl.

Agreement level	Conventional meat	Raw meat	Vegan	Total
Strongly disagree	137	59	23	219
Disagree	243	123	46	412
Neither agree nor disagree	238	145	56	439
Agree	389	212	97	698
Strongly agree	249	213	61	523
Total	1256	752	283	2291

10. Guarded food

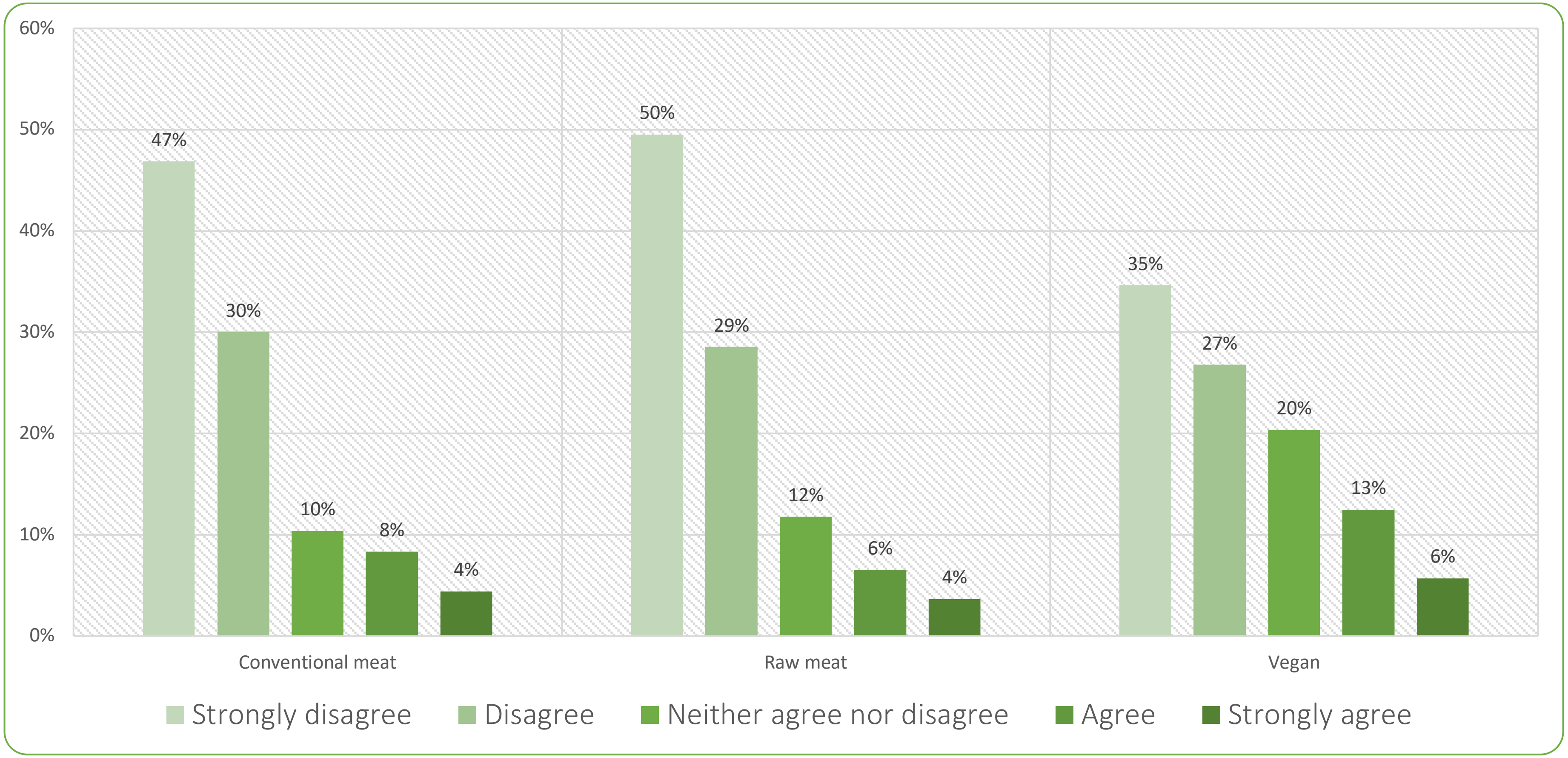

Figure A10. Percentage of dogs who guarded their food.

Table A10. Numbers of dogs who guarded their food.

Agreement level	Conventional meat	Raw meat	Vegan	Total
Strongly disagree	573	366	97	1036
Disagree	367	211	75	653
Neither agree nor disagree	127	87	57	271
Agree	102	48	35	185
Strongly agree	54	27	16	97
Total	1223	739	280	2242
