# Supplementary material for: Vegan versus meat-based pet foods: Owner-reported palatability behaviours and implications for canine and feline welfare
Source: PLoS One. 2021 Jun 16;16(6):e0253292. doi: 10.1371/journal.pone.0253292 (PMC8208530; doi:10.1371/journal.pone.0253292)
Supplement: S2 Appendix — (PDF) [file pone.0253292.s002.pdf]

S2 Appendix- Feline palatability indicators (15)

---

1. Rapid Approach
2. Vocalisations
3. Eating Quickly
4. Stays Near Bowl
5. Guards Food
6. Flick Ears
7. Flick Tail
8. Licking Lips
9. Licking Nose
10. Licking Food
11. Licking Bowl
12. Sniff/Investigate
13. Drop Food
14. Leaving Food Uneaten
15. Grooming

Nb: Lack of reporting in some cases reduced total numbers, in some following Tables and Figures.

1. Approached meals rapidly

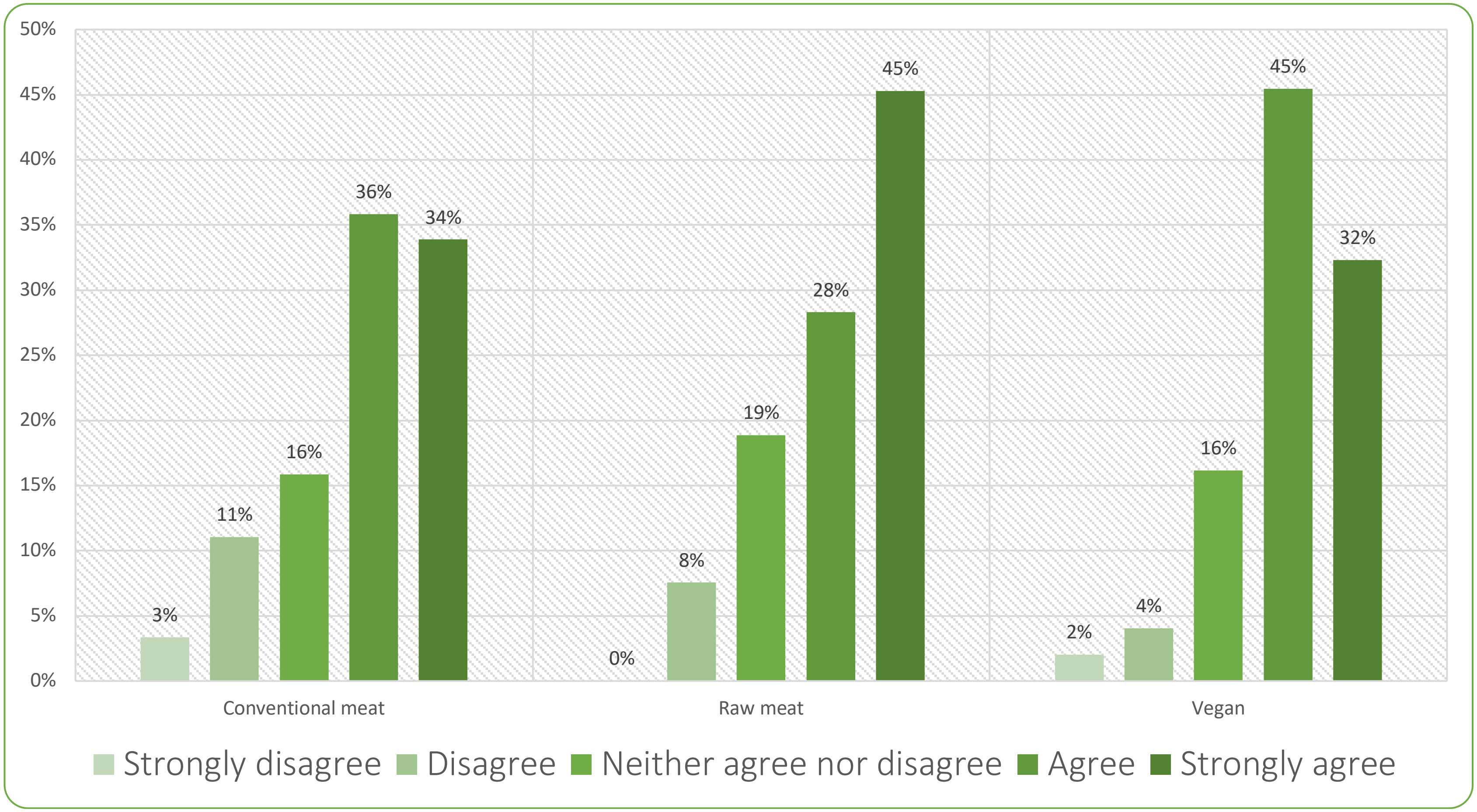

Figure B1. Percentage of cats who approached their meals rapidly.

Table B1. Numbers of cats who approached their meals rapidly.

Agreement level	Conventional meat	Raw meat	Vegan	Total
Strongly disagree	33		2	35
Disagree	108	4	4	116
Neither agree nor disagree	155	10	16	181
Agree	350	15	45	410
Strongly agree	331	24	32	387
Total	977	53	99	1129

2. Meowed/vocalised

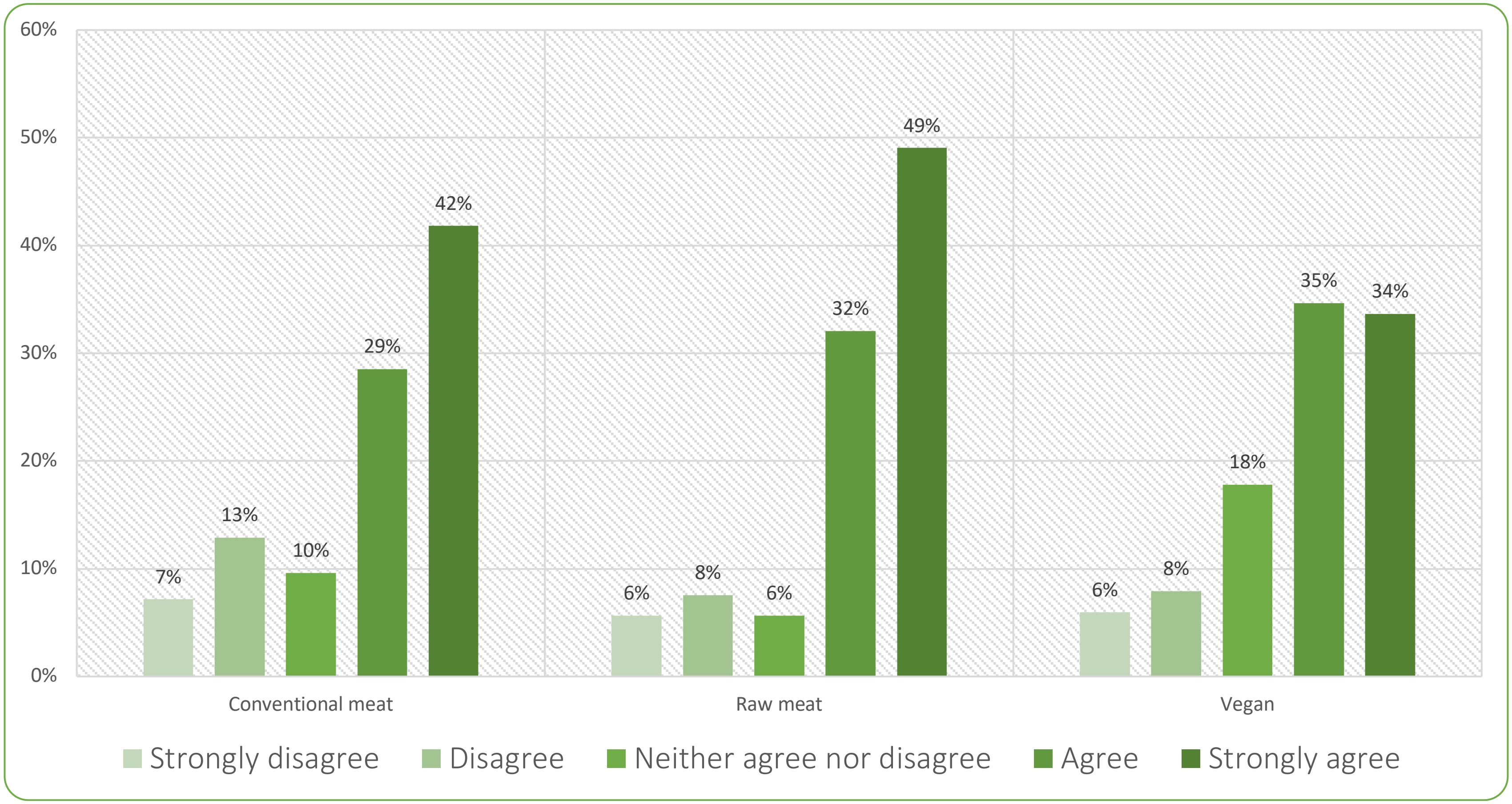

Figure B2. Percentage of cats who meowed/vocalised

Table B2. Numbers of cats who meowed/vocalised.

Agreement level	Conventional meat	Raw meat	Vegan	Total
Strongly disagree	70	3	6	79
Disagree	126	4	8	138
Neither agree nor disagree	94	3	18	115
Agree	279	17	35	331
Strongly agree	409	26	34	469
Total	978	53	101	1132

3. Ate quickly

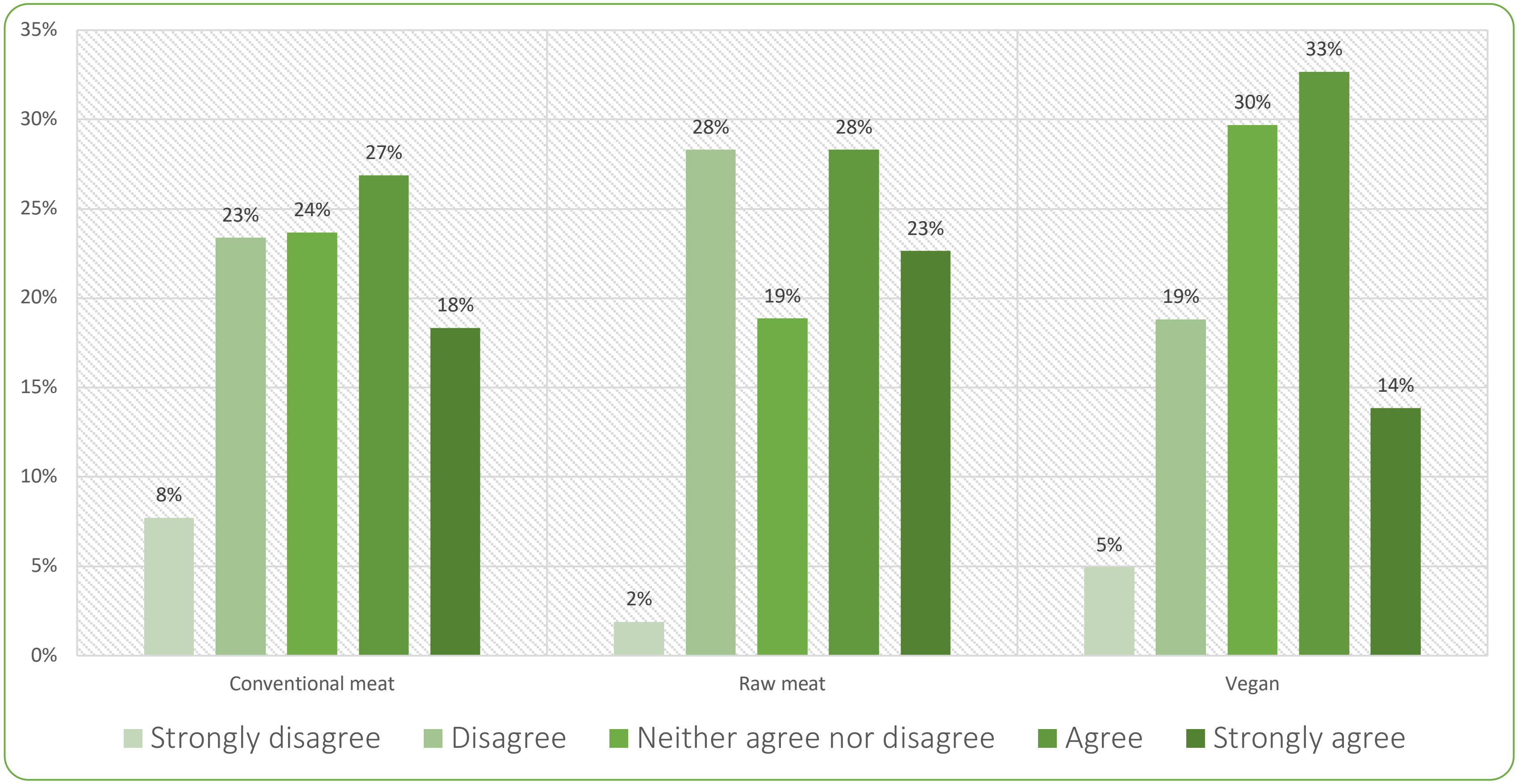

Figure B3. Percentage of cats who ate quickly.

Table B3. Numbers of cats who ate quickly.

Agreement level	Conventional meat	Raw meat	Vegan	Total
Strongly disagree	75	1	5	81
Disagree	227	15	19	261
Neither agree nor disagree	230	10	30	270
Agree	261	15	33	309
Strongly agree	178	12	14	204
Total	971	53	101	1125

4. Remained near food bowls

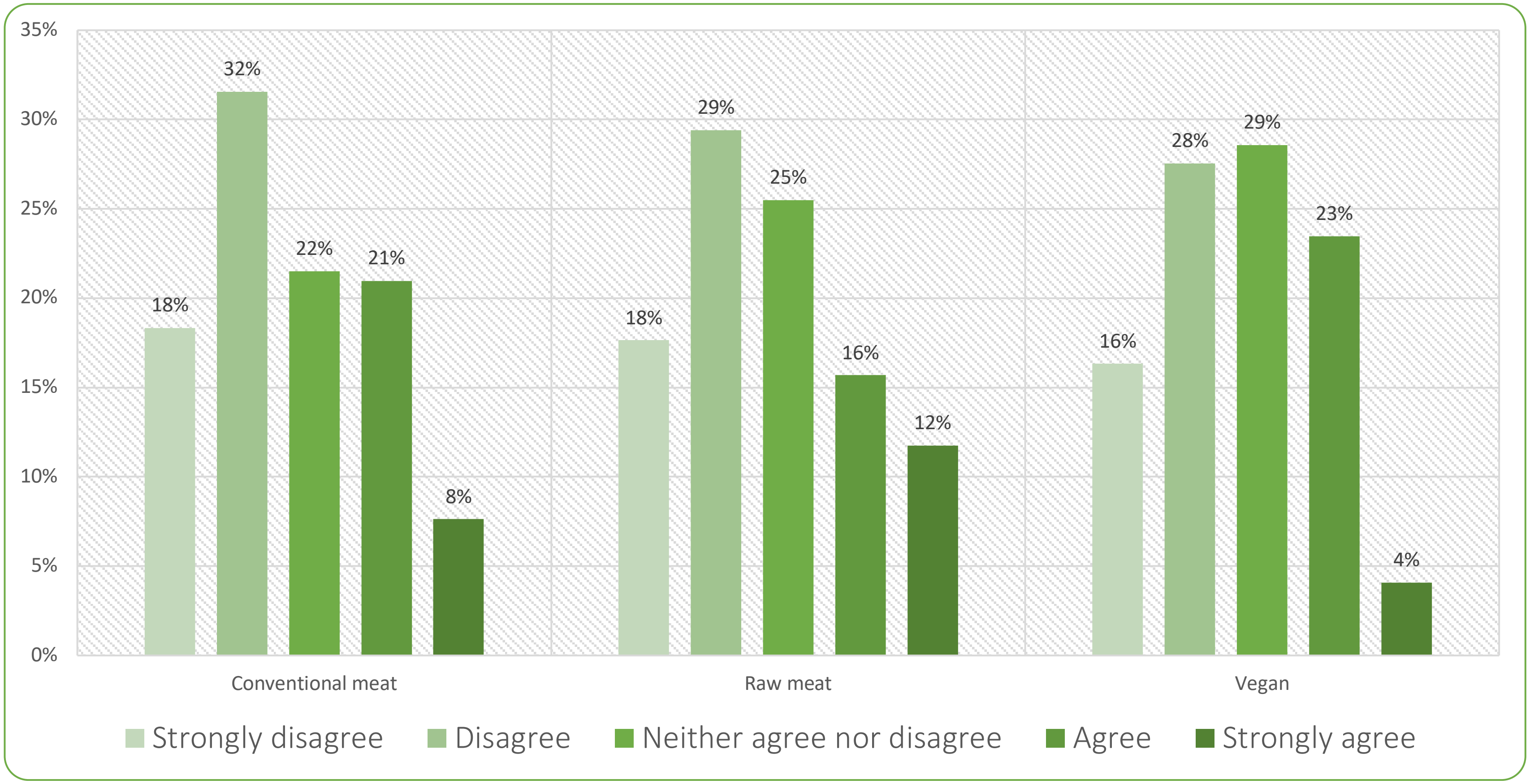

Figure B4. Percentage of cats who remained near their food bowls.

Table B4. Numbers of cats who remained near their food bowls.

Agreement level	Conventional meat	Raw meat	Vegan	Total
Strongly disagree	173	9	16	198
Disagree	298	15	27	340
Neither agree nor disagree	203	13	28	244
Agree	198	8	23	229
Strongly agree	72	6	4	82
Grand	944	51	98	1093

5. Guarded food

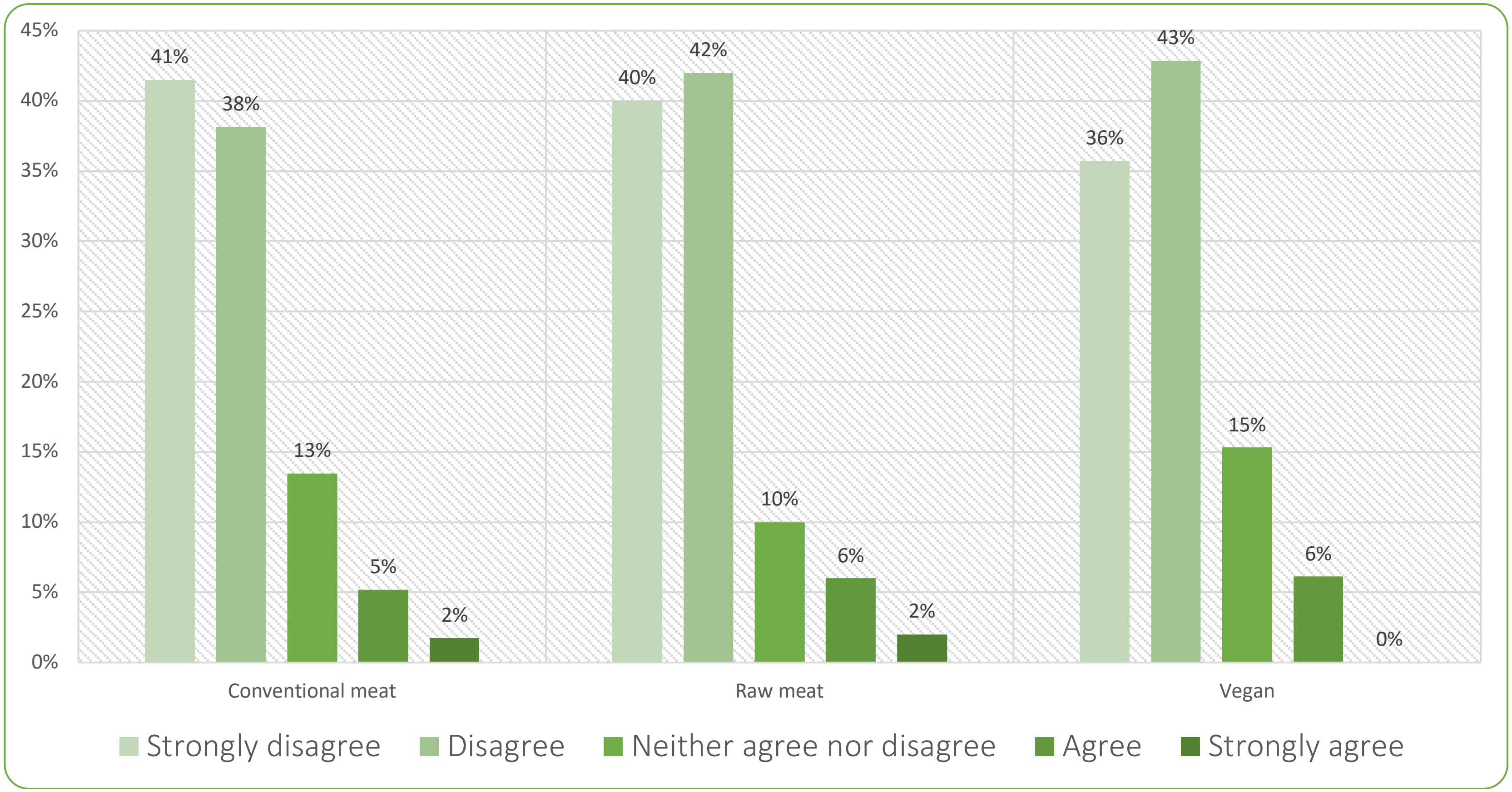

Figure B5. Percentage of cats who guarded their food.

Table B5. Numbers of cats who guarded their food.

Agreement level	Conventional meat	Raw meat	Vegan	Total
Strongly disagree	385	20	35	440
Disagree	354	21	42	417
Neither agree nor disagree	125	5	15	145
Agree	48	3	6	57
Strongly agree	16	1		17
Total	928	50	98	1076

6. Flicked ears backwards

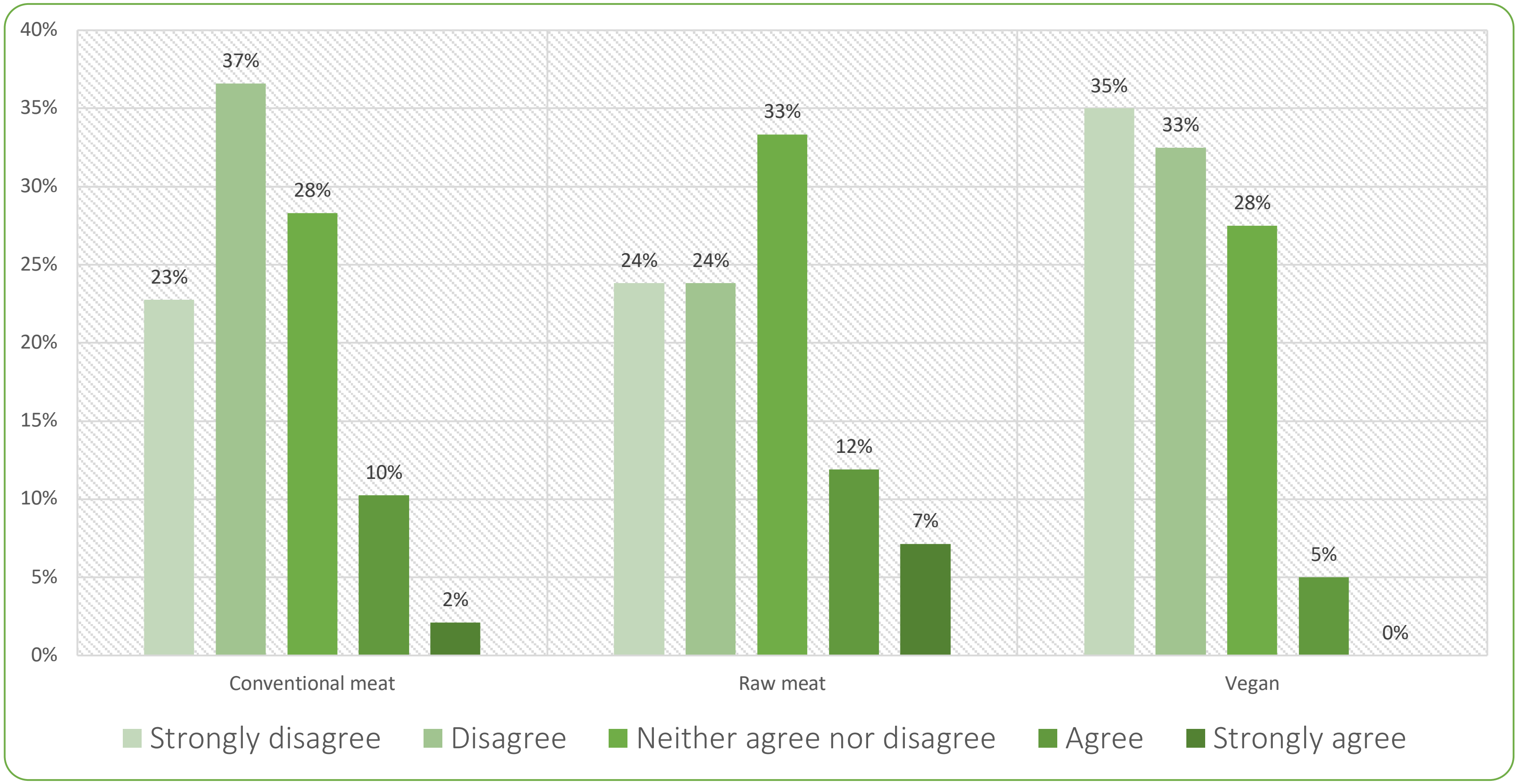

Figure B6. Percentage of cats who flicked their ears backwards.

Table B6. Numbers of cats who flicked their ears backwards.

Agreement level	Conventional meat	Raw meat	Vegan	Total
Strongly disagree	184	10	28	222
Disagree	296	10	26	332
Neither agree nor disagree	229	14	22	265
Agree	83	5	4	92
Strongly agree	17	3		20
Total	809	42	80	931

7. Flicked tails

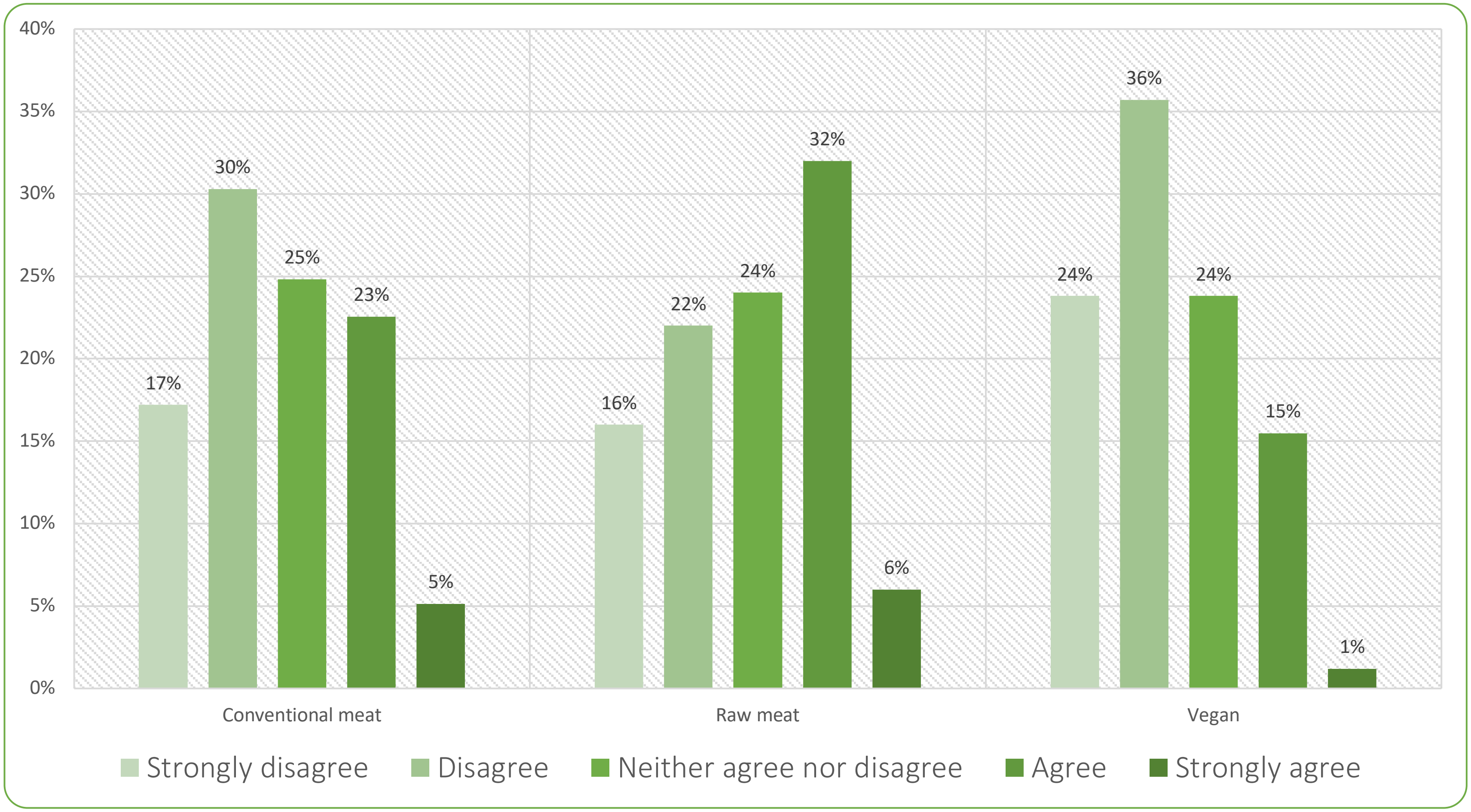

Figure B7. Percentage of cats who flicked their tails.

Table B7. Numbers of cats who flicked their tails.

Agreement level	Conventional meat	Raw meat	Vegan	Total
Strongly disagree	151	8	20	179
Disagree	266	11	30	307
Neither agree nor disagree	218	12	20	250
Agree	198	16	13	227
Strongly agree	45	3	1	49
Total	878	50	84	1012

8. Licked lips

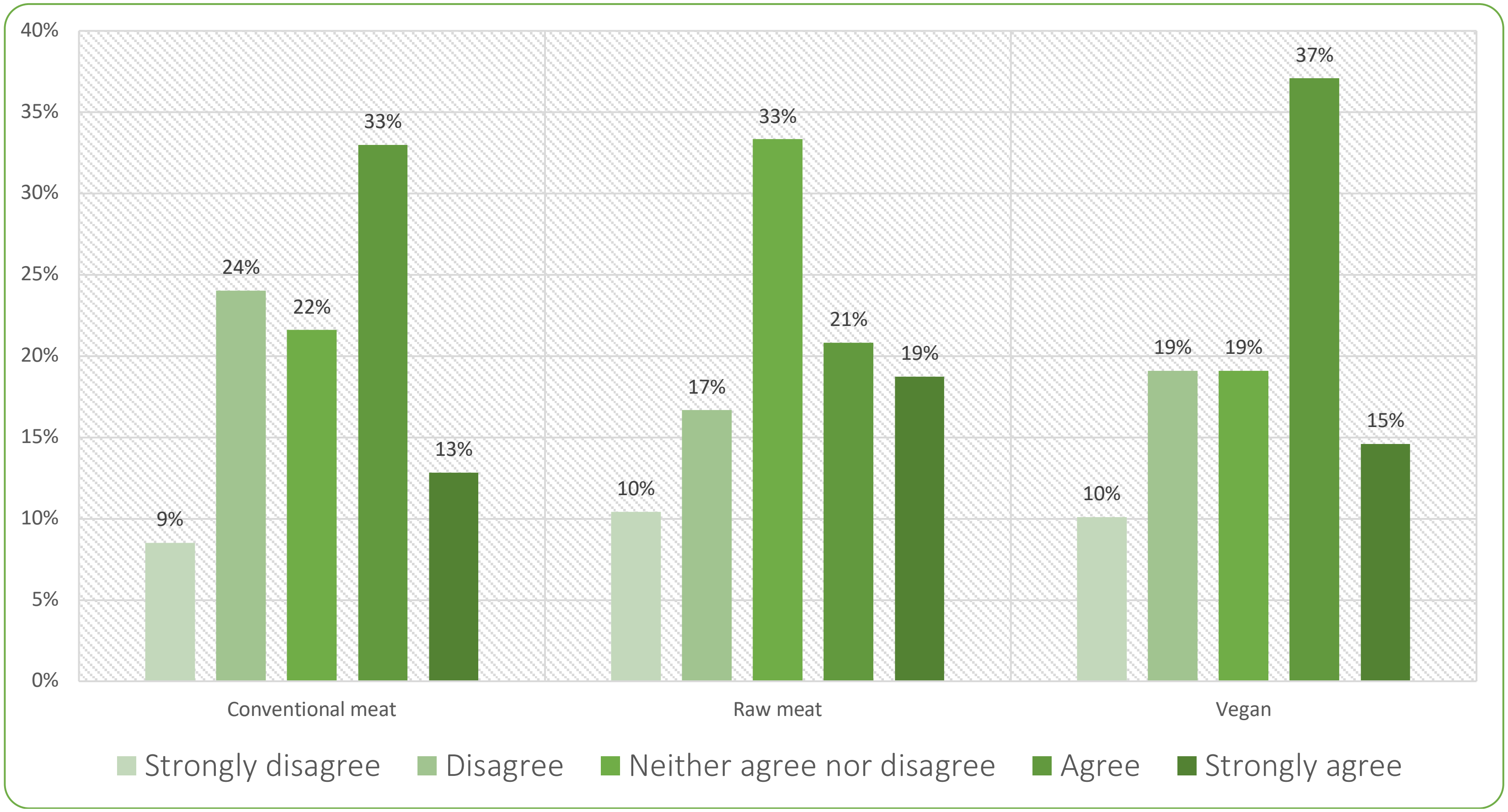

Table B8. Numbers of cats who licked their lips.

Table B8. Numbers of cats who licked their lips.

Agreement level	Conventional meat	Raw meat	Vegan	Total
Strongly disagree	77	5	9	91
Disagree	217	8	17	242
Neither agree nor disagree	195	16	17	228
Agree	298	10	33	341
Strongly agree	116	9	13	138
Total	903	48	89	1040

9. Licked noses

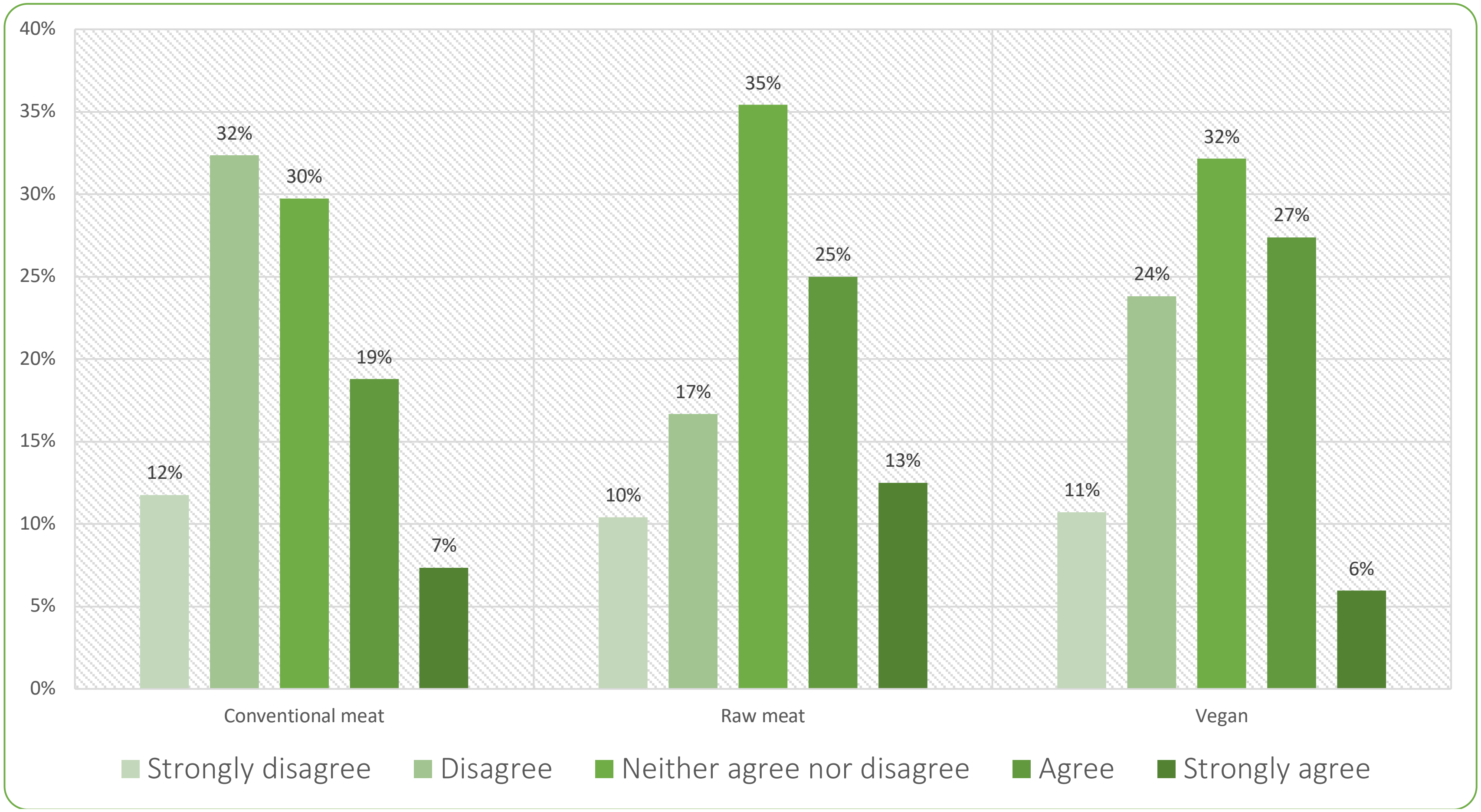

Figure B9. Percentage of cats who licked their noses.

Table B9. Numbers of cats who licked their noses.

Agreement level	Conventional meat	Raw meat	Vegan	Total
Strongly disagree	104	5	9	118
Disagree	286	8	20	314
Neither agree nor disagree	263	17	27	307
Agree	166	12	23	201
Strongly agree	65	6	5	76
Total	884	48	84	1016

10. Licked food

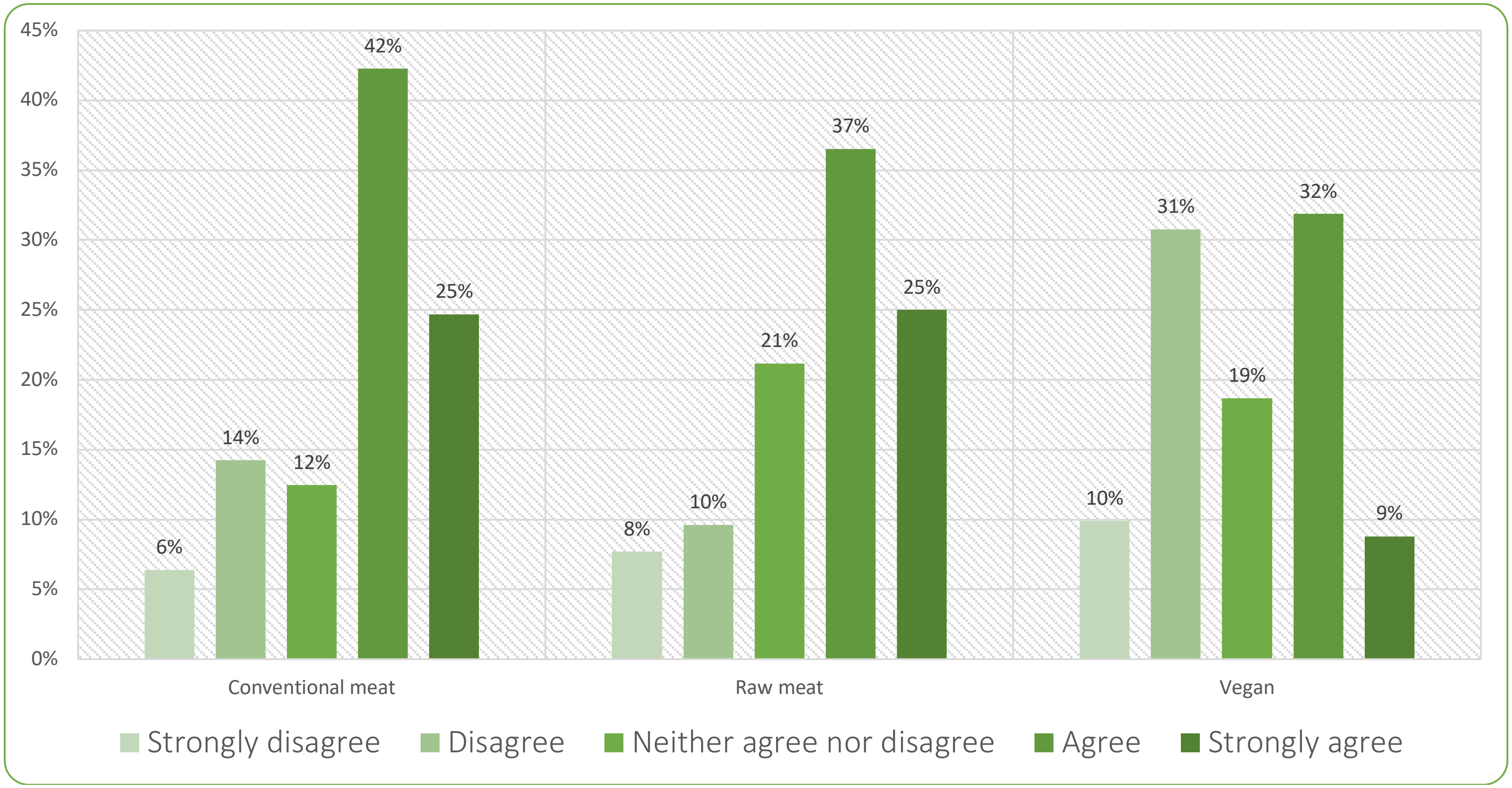

Figure B10. Percentage of cats who licked their food.

Table B10. Numbers of cats who licked their food.

Agreement level	Conventional meat	Raw meat	Vegan	Total
Strongly disagree	61	4	9	74
Disagree	136	5	28	169
Neither agree nor disagree	119	11	17	147
Agree	404	19	29	452
Strongly agree	236	13	8	257
Total	956	52	91	1099

11. Licked food bowls

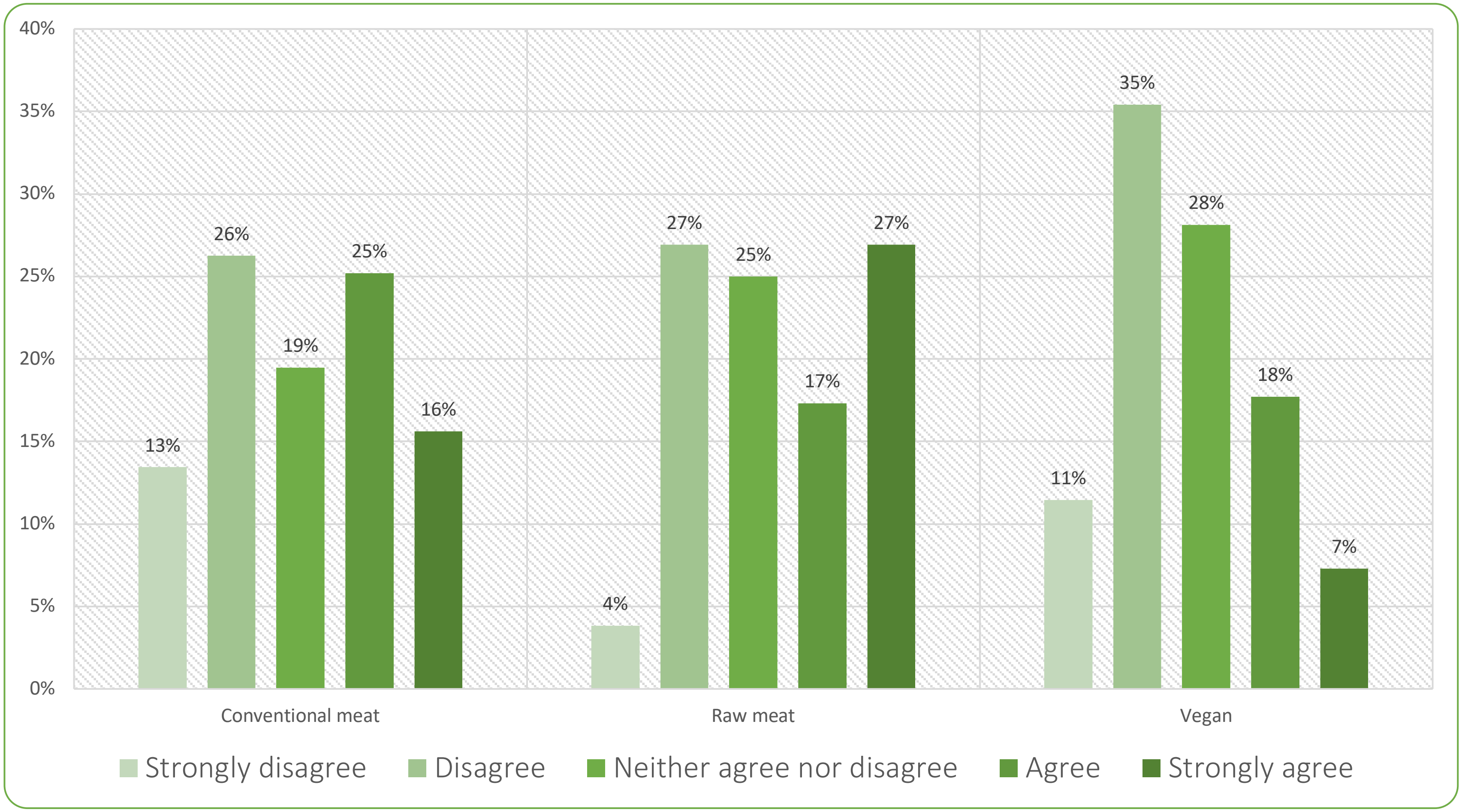

Figure B11. Percentage of cats who licked their food bowls.

Table B11. Numbers of cats who licked their food bowls.

Agreement level	Conventional meat	Raw meat	Vegan	Total
Strongly disagree	129	2	11	142
Disagree	252	14	34	300
Neither agree nor disagree	187	13	27	227
Agree	242	9	17	268
Strongly agree	150	14	7	171
Total	960	52	96	1108

12. Sniffed/investigated food

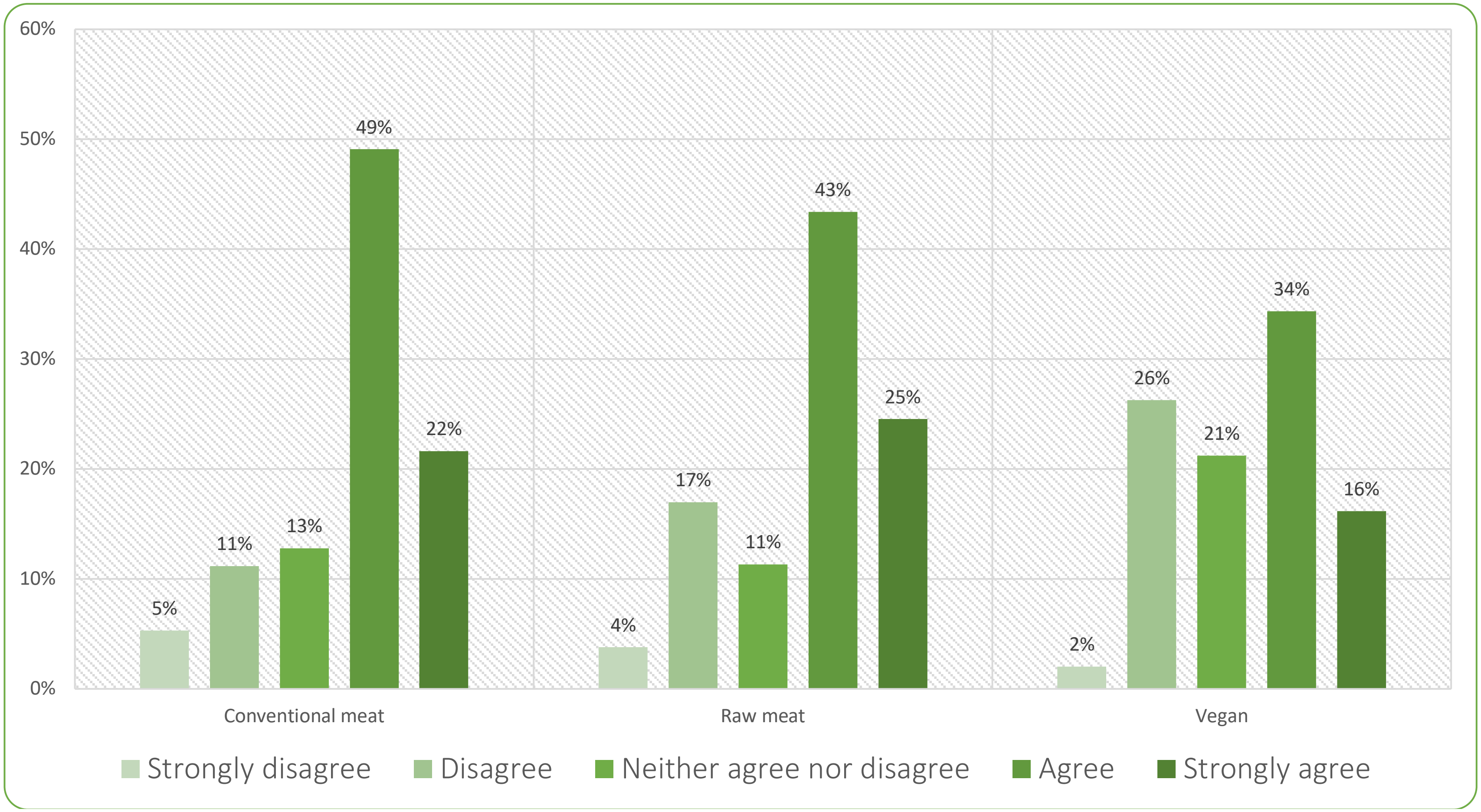

Figure B12. Percentage of cats who sniffed/investigated their food.

Table B12. Numbers of cats who sniffed/investigated their food.

Agreement level	Conventional meat	Raw meat	Vegan	Total
Strongly disagree	52	2	2	56
Disagree	109	9	26	144
Neither agree nor disagree	125	6	21	152
Agree	479	23	34	536
Strongly agree	211	13	16	240
Total	976	53	99	1128

13. Dropped food

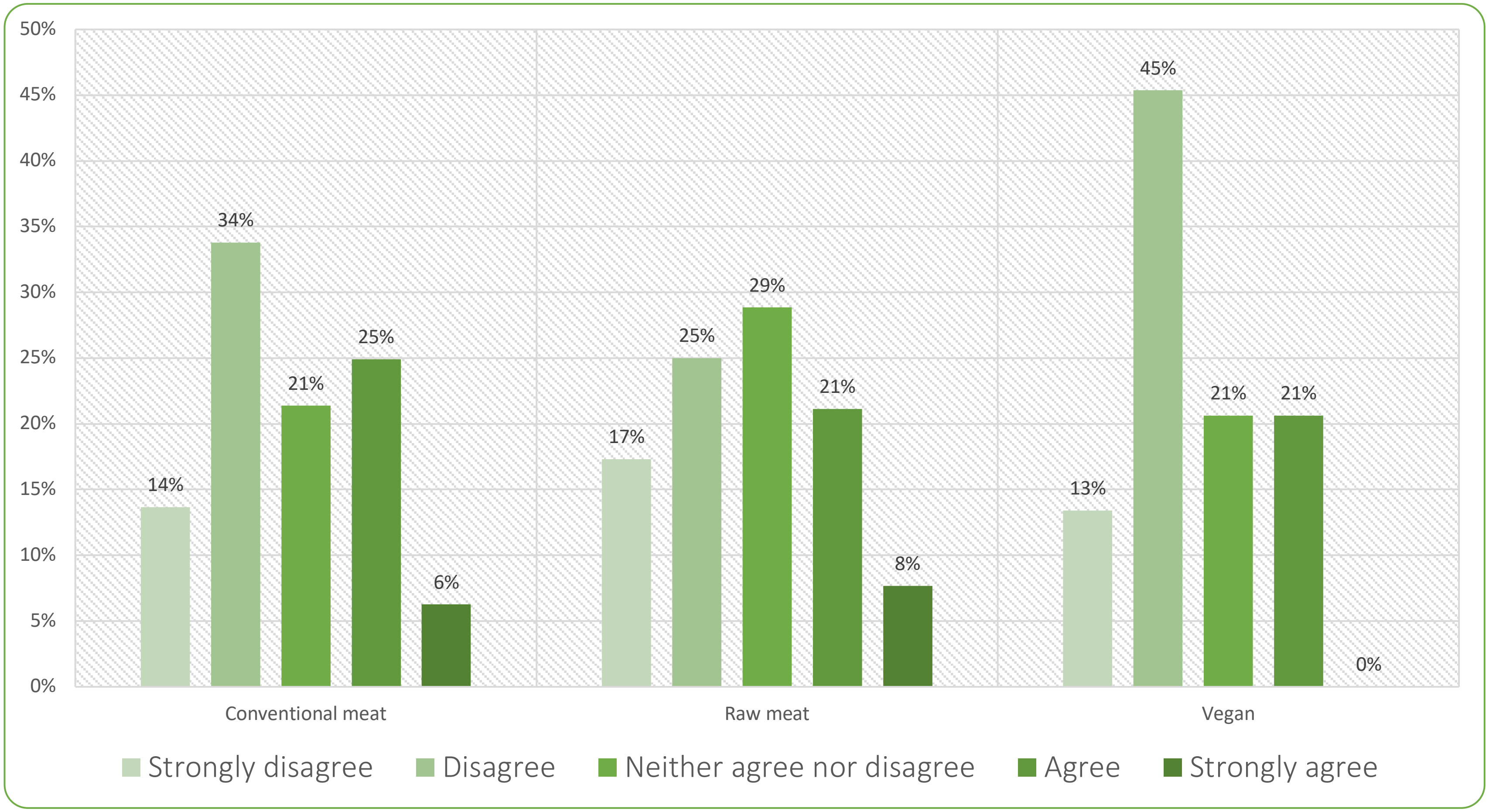

Figure B13. Percentage of cats who dropped their food.

Table B13. Numbers of cats who dropped their food.

Agreement level	Conventional meat	Raw meat	Vegan	Total
Strongly disagree	131	9	13	153
Disagree	324	13	44	381
Neither agree nor disagree	205	15	20	240
Agree	239	11	20	270
Strongly agree	60	4		64
Total	959	52	97	1108

14. Left food uneaten

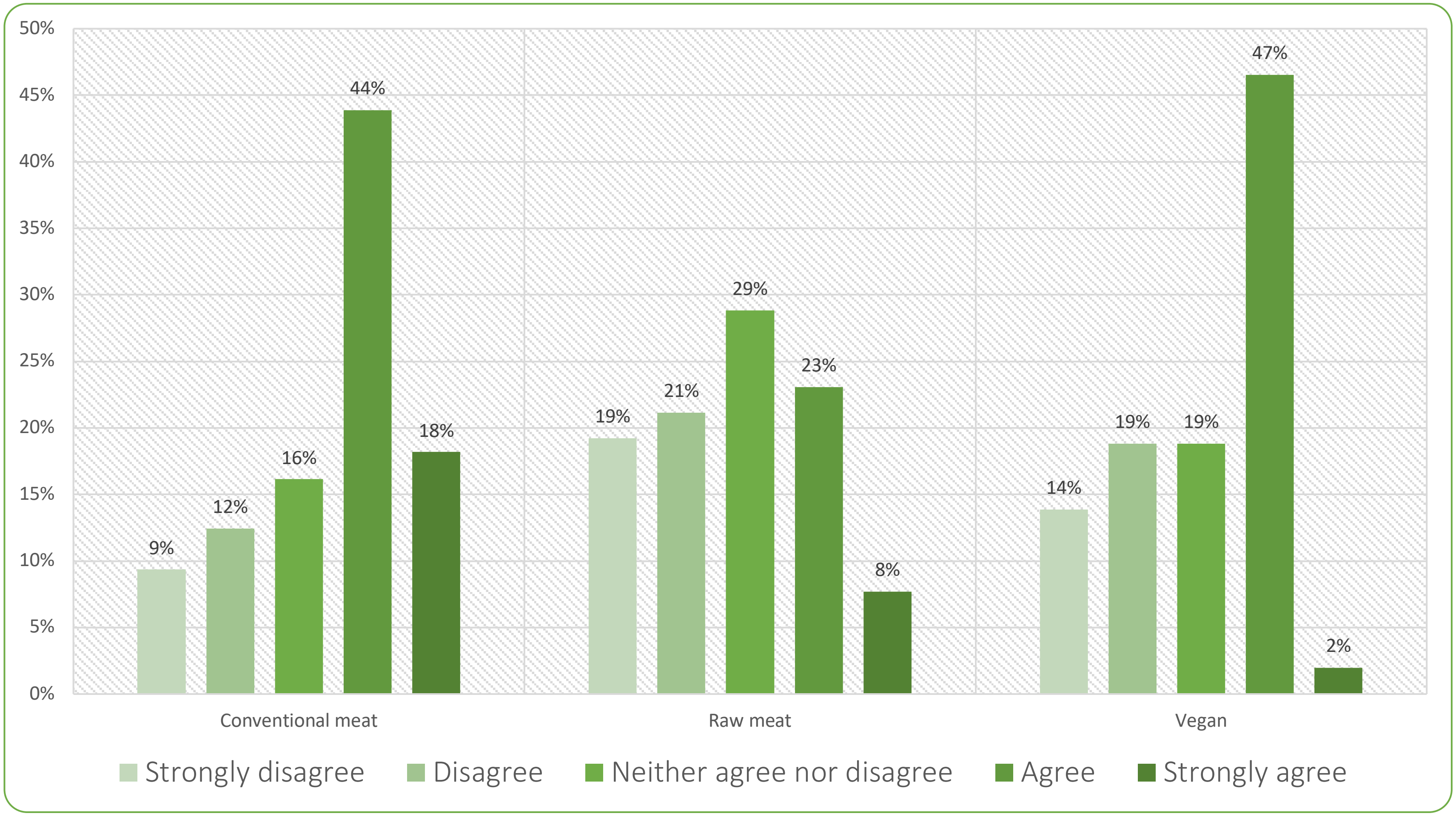

Figure B14. Percentage of cats who left food uneaten.

Table B14. Numbers of cats who left food uneaten.

Agreement level	Conventional meat	Raw meat	Vegan	Total
Strongly disagree	91	10	14	115
Disagree	121	11	19	151
Neither agree nor disagree	157	15	19	191
Agree	427	12	47	486
Strongly agree	177	4	2	183
Total	973	52	101	1126

15. Grooming

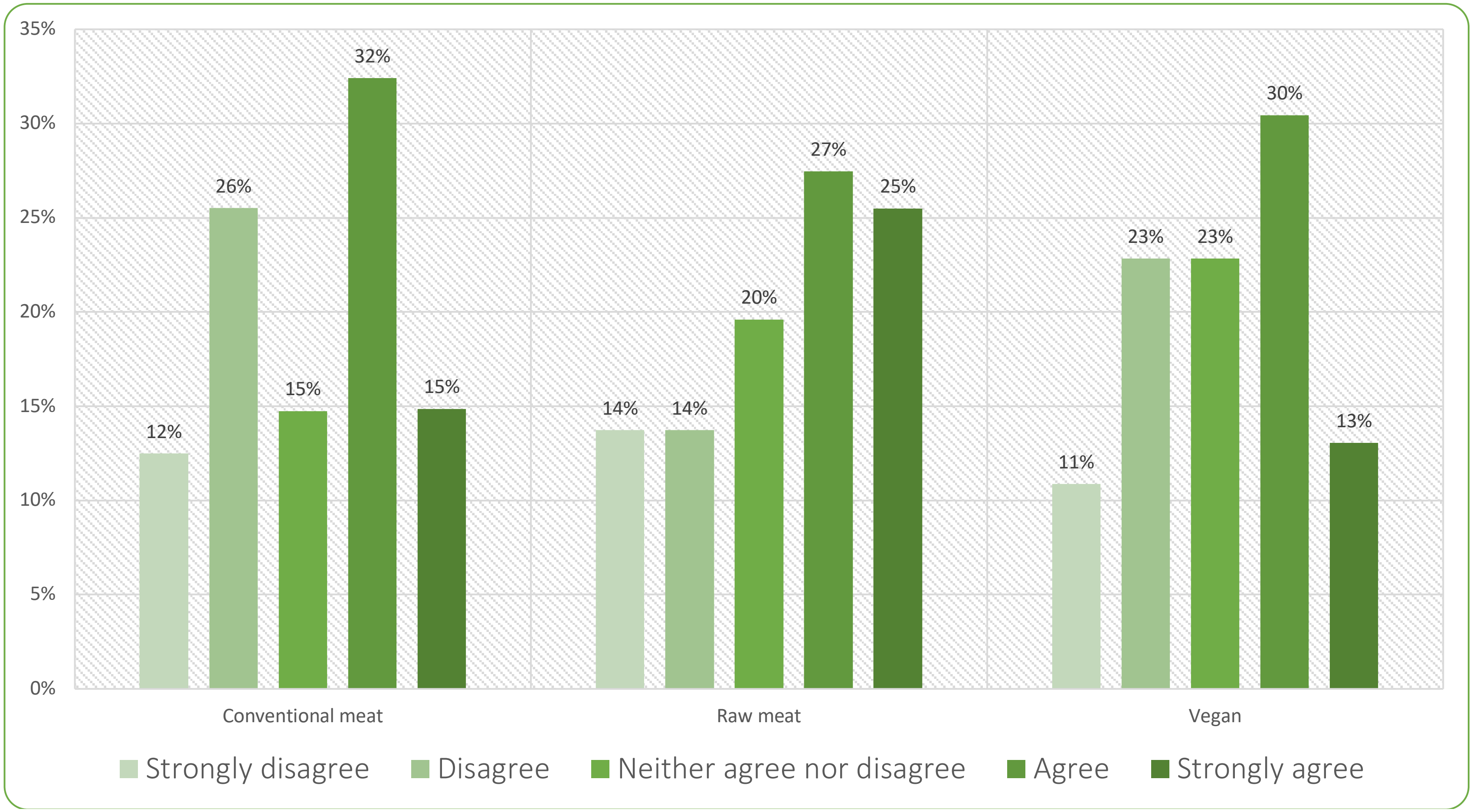

Figure B15. Percentage of cats who groomed themselves.

Table B15. Numbers of cats who groomed themselves.

Agreement level	Conventional meat	Raw meat	Vegan	Total
Strongly disagree	116	7	10	133
Disagree	237	7	21	265
Neither agree nor disagree	137	10	21	168
Agree	301	14	28	343
Strongly agree	138	13	12	163
Total	929	51	92	1072
